# Genome wide association analysis for grain micronutrients and anti-nutritional traits in mungbean [*Vigna radiata* (L.) R. Wilczek] using SNP markers

**DOI:** 10.3389/fnut.2023.1099004

**Published:** 2023-02-07

**Authors:** Mayank Kumar Sinha, Muraleedhar S. Aski, Gyan Prakash Mishra, M. B. Arun Kumar, Prachi S. Yadav, Jayanti P. Tokas, Sanjeev Gupta, Aditya Pratap, Shiv Kumar, Ramakrishnan M. Nair, Roland Schafleitner, Harsh Kumar Dikshit

**Affiliations:** ^1^Division of Genetics, ICAR - Indian Council of Agricultural Research– Indian Agricultural Research Institute, New Delhi, India; ^2^Division of Seed Science and Technology, ICAR – Indian Agricultural Research Institute, New Delhi, India; ^3^Division of Biochemistry, Chaudhary Charan Singh Haryana Agricultural University, Hissar, India; ^4^Krishi Bhavan, Indian Council of Agricultural Research, New Delhi, India; ^5^Division of Crop Improvement, ICAR – Indian Institute of Pulses Research, Kanpur, India; ^6^International Center for Agricultural Research in the Dry Areas (ICARDA), New Delhi, India; ^7^World Vegetable Center, South and Central Asia, Hyderabad, India; ^8^World Vegetable Center Head Quarters (HQ), Tainan, Taiwan

**Keywords:** micronutrients, marker trait association, anti-nutrients, bio-fortification, tannins

## Abstract

Mungbean is an important food grain legume for human nutrition and nutritional food due to its nutrient-dense seed, liked palatability, and high digestibility. However, anti-nutritional factors pose a significant risk to improving nutritional quality for bio-fortification. In the present study, genetic architecture of grain micronutrients (grain iron and zinc concentration) and anti-nutritional factors (grain phytic acid and tannin content) in association mapping panel of 145 diverse mungbean were evaluated. Based on all four parameters genotypes PUSA 1333 and IPM 02-19 were observed as desired genotypes as they had high grain iron and zinc concentration but low grain phytic acid and tannin content. The next generation sequencing (NGS)-based genotyping by sequencing (GBS) identified 14,447 genome-wide SNPs in a diverse selected panel of 127 mungbean genotypes. Population admixture analysis revealed the presence of four different ancestries among the genotypes and LD decay of ∼57.6 kb kb physical distance was noted in mungbean chromosomes. Association mapping analysis revealed that a total of 20 significant SNPs were shared by both GLM and Blink models associated with grain micronutrient and anti-nutritional factor traits, with Blink model identifying 35 putative SNPs. Further, this study identified the 185 putative candidate genes. Including potential candidate genes *Vradi07g30190*, *Vradi01g09630*, and *Vradi09g05450* were found to be associated with grain iron concentration, *Vradi10g04830* with grain zinc concentration, *Vradi08g09870* and *Vradi01g11110* with grain phytic acid content and *Vradi04g11580* and *Vradi06g15090* with grain tannin content. Moreover, two genes *Vradi07g15310* and *Vradi09g05480* showed significant variation in protein structure between native and mutated versions. The identified SNPs and candidate genes are potential powerful tools to provide the essential information for genetic studies and marker-assisted breeding program for nutritional improvement in mungbean.

## Introduction

Mungbean is one of nearly 150 species in the *Vigna* genus, with 22 endemic to India and 16 to Southeast Asia. Africa, on the other hand, is home to the most species. Mungbean [*Vigna radiata* (L.) Wilczek] is a diploid legume with a genome size of 0.60 pg/1C (579 Mbp) and a genome size of 0.60 pg/1C (579 Mbp) (1). Mungbean is a warm-season legume that grows between 40 and 10 degrees north in the tropics and subtropics. India, China, Pakistan, Bangladesh, Sri Lanka, Thailand, Myanmar, Vietnam, Indonesia, Australia, and the Philippines are the top mungbean producers (2). India is the world leader in mungbean production, with 4.53 million hectares yielding 2.08 million tons of grain (AICRP on MULLaRP PC Report 2020–21).

The recommended dietary allowance (RDA), which for adult women is roughly 0.06 g day^–1^ with a low-iron-bioavailability (5%) diet and 0.02 g day^–1^ with a high-iron-bioavailability (15%) diet, is not met by a sizeable fraction of the population in underdeveloped nations (3). The most vulnerable are women of reproductive age and children. Anemia affects around 88 percent of pregnant women and 63 percent of children aged 5–14 years in South Asia [ACC/SCN, 2000; (4)]. Micronutrient deficiency in humans is referred to as “hidden hunger.” Bio-fortification is a genetic enhancement method that boosts mineral absorption while lowering anti-nutritive components and balances mineral concentrations in edible plant parts and seeds (5, 6). Iron deficiency is the most prevalent micronutrient problem worldwide. Iron deficiency reduces the amount of oxygen delivered to cells, resulting in tiredness, poor work performance, lowered immunity, and mortality (7).

Zinc is an essential nutrient for plant growth and production due to its participation in over 300 enzymes involved in the metabolism of glucose, DNA, protein synthesis and digestion, and bone development. Zinc deficiency can lead to stunted growth, skin blemishes, and an increased risk of infection (8). For male adults, the RDA for zinc is 0.011 g per day, while for female adults, it is 0.008 g per day.^1^ A typical main organic form of phosphorus (P) storage in plants is phytic acid (PA) (chemically called as myo-inositol hexaphosphoric acid). Because it is an effective chelator of positively charged cations, PA binds to nutritionally important mineral cations such as calcium, iron, and zinc, as well as inhibiting trypsin (9). Furthermore, humans and other non-ruminants lack the phytase enzyme, which prohibits them from digesting PA and excreting a large part of these salts.

Tannins are polyphenolic compounds with a molecular weight more than 500 kD that can form a complex with proteins (10–12). Tannins are classified into two groups based on their structure: condensed tannins and hydrolysable tannins. The majority of tannins are found in the seed coat, with only a few residues in the cotyledons (13, 14). Condensed tannins seem to bind proteins very tightly, lowering protein digestibility in pulses *in vitro* (15, 16). Tannins have the potential to bind with proteins and prevent them from being absorbed by the body. Because tannins are extremely reactive, processing alters their profiles and amounts in foods, potentially affecting their anti-oxidant activity and nutritional value (17).

Quantitative trait loci (QTL) analyses through genome-wide association studies (GWAS) using molecular markers and high throughput sequencing techniques can be used to identify the genes underlying nutritional (grain minerals, protein content, and anti-oxidant capacity) and anti-nutritional (phytic acid and tannins) traits (18). The GWAS studies have been reported in many other legume crops also (19). The two major methods for finding genes or QTLs are linkage mapping and association mapping based linkage disequilibrium (LD). The capacity to examine only two alleles at any given locus in biparental crosses and low mapping resolution are two major constraints of linkage mapping (20), whereas association mapping promises to overcome these limitations (21). Furthermore, association mapping uncovers QTLs, allowing to take advantage of natural variation and find beneficial genes in the genome using modern genetic tools.

In the present experiment, we have utilized single nucleotide polymorphism (SNP) markers discovered by sequencing of diverse mungbean germplasm and used to test nutritional and anti-nutritional attributes to characterize the diverse mungbean panel for grain micronutrient (Fe, Zn) concentration and anti-nutritional factors (Phytic acid, Tannins). To study the genetic diversity among mungbean genotypes using SNP markers. And to identify the linked SNPs with grain micronutrient concentration, tannins and phytic acid content using association mapping approach.

## Materials and methods

### Plant materials and experimental conditions

The grain micronutrient concentrations (Fe, Zn) and anti-nutritional components (Phytic acid, Tannins) were investigated using 145 different mungbean genotypes and 5 checks (Supplementary Table 1), which included released varieties, advanced breeding lines, and exotic germplasm lines from the World Vegetable Center. The experiment was carried out at the Indian Agricultural Research Institute (IARI) in New Delhi at the experimental field (28.638690, 77.156046). The genotypes were planted in the Augmented block design (22) with five checks in each 5 blocks. However, only one hundred and twenty-seven lines were selected for association mapping, as shown in Supplementary Table 2.

#### Iron and zinc estimation

The hand-threshed grains were then carefully placed in a clean plastic tray (using contaminant-free gloves). Each sample’s grains were rinsed in double distilled water and dried for 5 days at 35°C in a contamination-free, non-corroded oven. Using a mortar and pestle, 10 g of grains from each sample were manually ground into a fine powder. A microwave digestion device was used to digest the grain powder sample (1 g) according to the modified diacid technique (23). The Fe and Zn concentrations in three technical replications per biological sample (in mg/kg seed) were determined using an AAS (Atomic Absorption Spectrophotometer) (ElementAS, Electronics Corporation of India Ltd., Model- AAS4141).

#### Phytic acid estimation

Megazyme Phytic acid assay kit (2022 Megazyme Ltd.) was used to estimate phytic acid content. The total phosphate emitted is calculated as grams of phosphorus per 100 g of sample material using a modified colorimetric approach. Using a spectrophotometer with a wavelength of 655 nm, the total phosphate emitted during the procedure was measured.

#### Estimation of condensed tannin (pro-anthocyanidin) content (CTC)

The approach provided by de Camargo et al. (24) was used to evaluate condensed tannins in lentils.

### Statistical analysis

The data recorded on investigated traits were subjected to descriptive statistics including mean, standard deviation, range, coefficient of variation, and broad sense heritability. The analysis of variance (ANOVA), Pearson’s correlation analysis, cluster analysis and principal component analysis for recoded data were performed in R (Version 4.1.2). Frequency distribution graphs of recorded traits were developed using MS-EXCEL program. The ANOVA was carried out for the Augmented Block Design Using R agricolae package 1.4.0.

#### Genomic DNA extraction, purification, and quantification

According to Doyle and Doyle (25), genomic DNA was isolated from immature mungbean leaves using the CTAB (CetylTrimethyl Ammonium Bromide) method. The DNA was quantified using an agarose gel electrophoresis method. This was accomplished by dissolving 0.8 g of agarose powder in 100 ml of 1X TBE buffer to obtain a 0.8 percent final concentration of agarose. The DNA integrity was further evaluated using a double beam UV spectrophotometer at 260 and 280 nm wavelengths. The concentration of DNA was calculated using the following formula:


Concentration⁢of⁢ds⁢DNA=⁢50⁢μ⁢g/m⁢l×O⁢D⁢(260)⁢×dilution⁢factor.

The protein contamination of samples was determined using the optical density (OD) ratio at 260/280 nm. If the ratio is less than 1.8, the sample has been contaminated with protein. The ratio of pure DNA is 1.8.

#### Genome wide discovery of GBS based SNP markers

127 mungbean genotypes of AM panel, used for molecular characterization and association mapping (AM) (Supplementary Table 2).

Then samples are digested with APeKI restriction enzyme and ligated to adaptors with unique barcodes to create 127-plex GBS libraries, then pooled (26). The generated libraries were single end sequenced (150 bp) using the Illumina HiSeq 4000 NGS platform as per Bastien et al. (27) and Kujur et al. (28). The GBS assay’s repeatability was tested using a non-template control and biological duplicates with three accessions. For quality assessment of sequence reads, the resulting FASTQ sequence files were processed using the STACKS v1.0^2^ sliding window technique (29). Sequence readings with a quality of 90% below confidence were eliminated, as were sequence reads with long decreases in quality (30). The FASTQ sequence reads were mapped and aligned to the mungbean reference genome (31) using Bowtie v2.1.0 after demultiplexing using unique barcodes (32). Furthermore, the SNPs were accurately identified by processing the resulting SAM (sequence alignment map) files of 127 genotypes utilizing a reference based GBS technique of the STACKS v1.0 approach. The STACKS *de novo* based GBS technique was used to process the unmapped sequence reads on the reference genome yet again. The sampled SNPs were reconstructed into STACKS from sequencing reads of each genotype for the detection of probable SNPs, as described by Kujur et al. (28) and Hohenlohe et al. (33). Structure and functional annotation were carried out according to the mungbean genome (31) annotation to determine the precise position of GBS-based SNPs in different variations of the genome.

#### Molecular diversity and population structure

The indices representing molecular diversity including θπ (nucleotide diversity based on substitution of nucleotide in any two randomly selected DNA sequences at a particular site), θω (Watterson’s estimator of segregating sites, based on mutation rate estimates from loci that are segregating in population) and Tajima’s D (to test the null hypothesis of selective neutrality within the population) were estimated using a TASSEL v5.0 sliding window approach as suggested by Xu et al. (34) and Varshney et al. (35). The population structure among the 127 genotypes was identified by evaluating the obtained SNP data with ADMIXTURE version 1.3.0’s model-based program, utilizing (36) method. Furthermore, the *ad-hoc*, delta K technique was used to calculate the optimum population number (K) value, as described by Evanno et al. (37). The collected SNP data of 127 genotypes were examined using TASSEL v5.0 (38) software to construct an unrooted neighbor-joining (NJ)-based phylogenetic tree (with 1,000 bootstrap replicates).

#### Linkage disequilibrium (LD) measurement

The correlation between pairs of SNP sites on the chromosome is mostly determined by LD (39). As a result, the correct measurement of LD is the square of correlation (r^2^) between pairs of alleles (40). The degree of LD and its degradation in the population largely affected the identification of markers connected to trait loci and the resolution of association analyses (41). Several statistics for LD assessment were derived based on the effect of sample size and marginal allelic frequencies (42). To assess patterns of LD (r^2^) and LD decay, the produced SNP data were analyzed using TASSEL v5.0 (sliding window technique) and R (Version 4.1.2) in the current study [following Remington et al. (43)].

#### Association mapping (AM) of investigated traits

Using the HapMap file including genotypic data for 127 different genotypes as well as phenotypic data, GAPIT (Genomic Association and Prediction Integrated Tool) was utilized to run a GWAS on seed Iron and Zinc concentration as well as Phytic acid and Tannin content. GAPIT (version 3) was used to conduct GAWAS, which used MLM and BLINK models. BLINK (Bayesian-information and Linkage-disequilibrium Iteratively Nested Keyway) is a Genome Wide Association Study (GWAS) Method (44). (BLINK). BLINK stands for “Fixed and random model Circulating Probability Unification” and is an improved version of the FarmCPU GWAS approach. BLINK uses a multi-locus model for evaluating markers across the genome, similar to the Multi-loci Mixed Linear Model (MLMM). BLINK iteratively runs two fixed effect models. To account for population stratification, one model tests each marker one at a time, with many associated markers fitted as covariates. The other model uses covariate markers instead of kinship to directly control spurious association, removing the confounding between testing marker and kinship. To boost statistical power, BLINK eliminates the requirement that genes underlying a characteristic be scattered evenly across the genome. To improve processing speed, BLINK substitutes the REstricted Maximum Likelihood (REML) in a mixed linear model with Bayesian Information Content (BIC) in a fixed effect model in FarmCPU. The first three main components produced from all of the markers, as well as the origin-group, are included in the covariate variables. To eliminate linear dependency, the origin group was coded as an indicator (0/1) for each of the origin groups except the last one. The default GAPIT settings were utilized, as well as a Bayesian Information Criterion (BIC) model selection, which determines the degree of population structure that should be accounted for in a model to minimize overfitting. According to the BIC analysis, none of the models required the use of PCs. To account for population stratification, a mixed linear model with a kinship matrix was chosen for analysis. The following formula was used to fit the mixed linear models:


y=X⁢β+Z⁢μ+e

According to the GAPIT user manual, y is a vector of observed phenotypes, b is an unknown vector containing fixed effects that account for the genetic marker, population structure (Q), and intercept, u is an unknown vector of random additive effects from background QTLs and individuals, X and Z are the known design matrices, and e is an unobserved vector of residuals. For GWAS of nutritional traits, the Bayesian-information and Linkage-disequilibrium Iteratively Nested Keyway (BLINK) method was used because it has high statistical power and does not assume that causal genes are distributed normally across the genome, which can lead to false positives and exclusion of causal genes (45). Only the most important markers are reported since BLINK utilizes BIC to eliminate markers based on linkage disequilibrium (LD) (45). The following formula was used to suit the BLINK models:


$$y=s_{i}+S+e$$

Where y is a vector of observed phenotypes; s_*i*_ is a testing marker; S is a pseudo quantitative trait nucleotide (QTN), and e is the unobserved vector of residuals according to the GAPIT user manual. A Bonferroni correction was used to avoid false positives and identify significant SNPs (a^1/4^0.05) for each trait. The Bonferroni correction was calculated as −log_10_ (0.05/n), where n equals the number of SNPs used in the GWAS for each trait.

The threshold of significance, threshold probability of –log 10 (*p*-value) > 3.0 was used as cut off to identify significant markers associated with grain iron and grain zinc concentration while threshold probability of –log 10 (*p*-value) > 4.0 was used as cut off to identify significant markers associated with grain phytic acid and grain tannin content. For multiple comparisons, the significance threshold of the adjusted *p*-value was corrected according to the false discovery rate (FDR) with cut off ≤ 0.05 (46). The *p*-value distribution of significant SNP markers related with examined attributes was depicted using Manhattan plots. The adequacy of controlling type I error was assessed by plotting observed and expected –log10 (p) values using quantile-quantile (Q-Q) plots following Diapari et al. (47).

#### Delineation of putative candidate genes for investigated traits

Initially, genes with SNP variations were identified by functional annotation with the mungbean reference genome to identify the likely candidate genes affecting the characteristics in the study (31). A window of 57,679 kb in the vicinity of SNPs in the genomic area was investigated to identify candidate genes affecting the attributes. The interval sequences were retrieved and mapped on the mungbean genome using the mungbean reference genome (31), and the candidate genes were found using the reference genome location generated by blast. The SNPs within respective LD decay range of respective chromosome were considered as the same locus and SNP sites were considered as significantly associated. Following on, a legume information system^3^ was used to retrieve the CDS sequences of all protein-coding genes. SNPs found in the CDS section of prospective candidate genes were evaluated for type of SNP using TASSEL software, and the matching CDS sequences were translated to their expressed amino acid sequences using the EXPASY website’s facilities.^4^ In the I-TASSER platform,^5^ the differing amino acid sequences acquired from EXPASY were utilized to estimate protein structure (48).

## Results

The ANOVA augmented block design revealed the presence of highly significant variation among the genotypes for all tested traits (Supplementary Table 3). The study revealed the highly significant interaction between iron and phytic acid content being negatively correlated. Among the studied traits, the coefficient of variation was 21.23% for grain iron concentration, 29.90% for grain zinc concentration, 28.80 for grain phytic acid content and 26% for grain tannin content. The mean values obtained for iron was 74.15 mg Kg^–1^, for zinc it was 32.20 mg Kg^–1^, for phytic acid it was 7.35 mg g^–1^ and for tannins the value was 3.8 g 100 g^–1^) (Supplementary Table 4).

The genotypes showed variation for iron content in the range of 48.2–121.85 mg Kg^–1^ where genotypes GANGA 8 (121.85 mg Kg^–1^), ML 818 (121.20 mg Kg^–1^), KM 16–69 (114.20 mg Kg^–1^) showed highest iron content. The zinc content for which the values ranged from 8.6 to 61.05 mg Kg^–1^. The genotypes BASANTI (61.05 mg Kg^–1^), IC 325828 (59.45 mg Kg^–1^), KM 16–75 (52.25 mg Kg^–1^) showed highest zinc content. The phytic acid values ranged from 1.5 to 14.85 mg g^–1^ in the genotypes. Some of the lowest phytic acid values were observed in the genotypes IPM 02–19 (1.5 mg g^–1^), GANGA 8 (3 mg g^–1^), PUSA 1333 (3.8 mg g^–1^), M1209 (4.19 mg g^–1^), IPM 02–14 (4.4 mg g^–1^), MH 1442 (4.46 mg g^–1^), and IC 436637 (4.7 mg g^–1^). Considerable variations were also seen in the tannin content in the studied genotypes which varied from 2.14 g 100 g^–1^ to 6.25 g 100 g^–1^. IPM 288 (2.14 g 100 g^–1^), PUSA 1333 (2.33 g 100 g^–1^), KM 2241 (2.33 g 100 g^–1^) showed the lowest values for the tannin content (Supplementary Table 5).

### Frequency distribution, genetic correlations, principal component analysis, and cluster analysis

**FIGURE 1:**
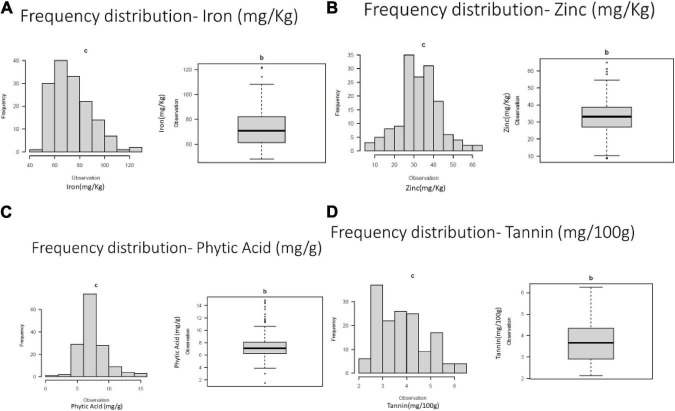
Frequency distribution of variation for **(A)** grain iron concentration **(B)** grain zinc concentration **(C)** grain phytic acid content and **(D)** grain tannin content.

Frequency distribution of variation for studies traits were presented in Figure 1.

**FIGURE 2:**
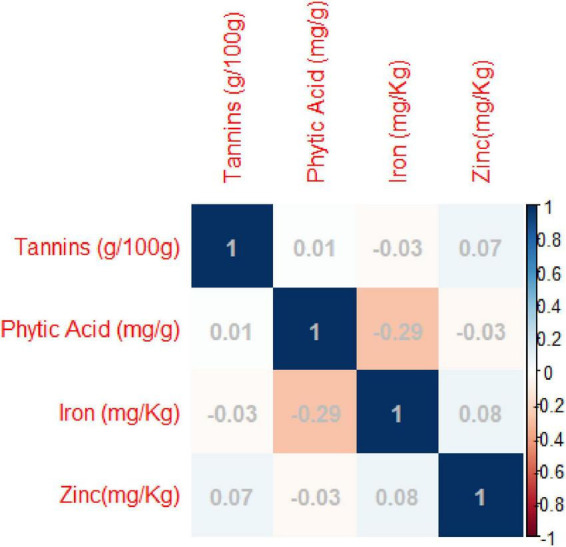
Pearson’s correlation coefficients between investigated traits- Diagrammatic view PC1 = 32.95%, PC2 = 27.60%.

Pearson’s correlation coefficients indicated significant negative correlations between grain iron concentration and grain phytic acid content. While non-significant negative correlation was observed between grain zinc concentration and grain phytic acid content and grain phytic acid content and grain tannin content (Supplementary Table 6 and Figure 2).

**FIGURE 3:**
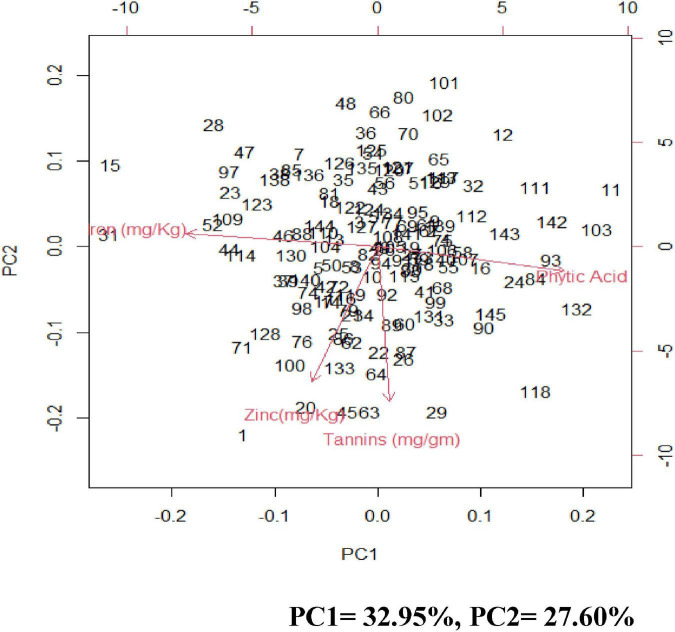
Biplots of the first two principal components (PC) showing variation among grain iron concentration, grain zinc concentration, grain phytic acid content, and grain tannin content.

Principal component analysis (PCA) was carried out to identify the most contributing traits of variation in the studied genotypes. The first principal component explained 32.95% of total variation, the second principal component explained 27.60% of total variation, the third principal component explained 21.9% of total variation and the fourth principal component explained 17.55 and all totaling 100% of total variation (Supplementary Table 7 and Figure 3).

### The genetic diversity among mungbean genotypes using SNP markers

The reference genome based GBS approach resulted in the detection of 14,447 high quality SNPs with a read depth of 10, 0% missing data, 10% heterozygosity, and 1% minor allele frequency (MAF).

Among all the 11 chromosomes, the maximum number of SNPs was observed on chromosome 1 (2,437 SNPs), whereas the minimum number of SNPs was observed on chromosome 3 (652 SNPs). The average SNP density (SNPs per 50 kb) was found to be high on chromosome 1 (3.33) and low on chromosome 5 (1.5) (Table 1).

**TABLE 1:** Details of the number of SNPs and their distribution on 11 mungbean chromosomes.

Chromosome	Chromosome size (bp)	SNPs per chromosome	Average density (SNPs per 50 kb)
Chromosome 1	36,501,346	2,437	3.34
Chromosome 2	25,360,630	1,199	2.36
Chromosome 3	12,950,713	652	2.52
Chromosome 4	20,812,224	1,015	2.44
Chromosome 5	37,180,910	1,124	1.51
Chromosome 6	37,436,759	1,527	2.04
Chromosome 7	55,601,358	2,030	1.83
Chromosome 8	45,727,239	1,702	1.86
Chromosome 9	21,008,463	1,119	2.66
Chromosome 10	20,996,616	815	1.94
Chromosome 11	19,732,206	827	2.10
Total	**333,308,464**	**14,447**	**2.17**

### Molecular diversity and population structure analysis

**FIGURE 4:**
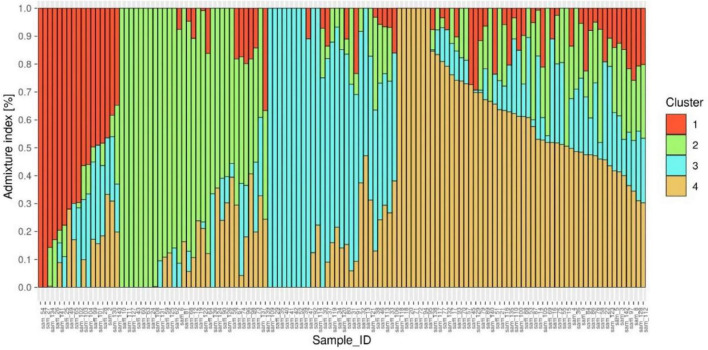
Population genetic structure plot of 127 diverse mungbean genotypes with optimal population number *k* = 4 with four different colors.

ADMIXTURE version 1.3.0. software (49) produces a Q matrix containing estimates of ancestry for each individual tested. The corresponding Q matrix with the lowest cross-validation error was chosen as the most representative of the study population, which was at K^1/4^4, corresponding to 4 distinct subpopulations (Figure 4 and Supplementary Figure 1).

The Q matrix was then sorted by the ancestry coefficients for each subpopulation, assigning individuals with coefficients > 50% to the corresponding sub population (50). These grouping of genotypes into three subpopulations were further confirmed by the distinct differentiation of genotypes into four clusters by unrooted neighbor-joining phylogenetic tree construction (Supplementary Figure 2).

#### Linkage disequilibrium analysis

**FIGURE 5:**
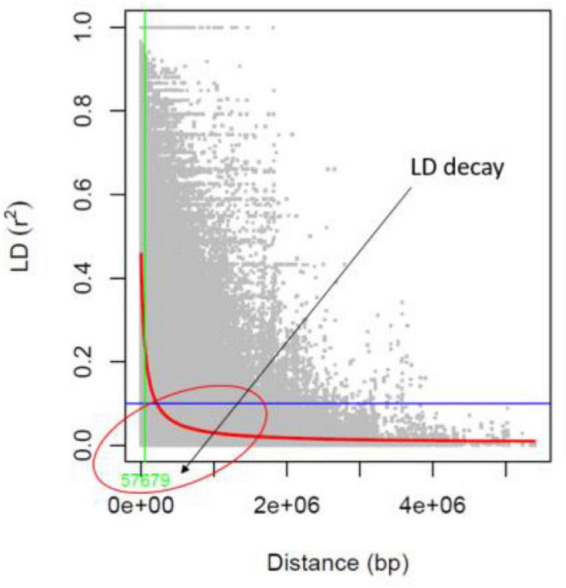
Linkage disequilibrium (LD) decay value (bp) in the association mapping panel.

The identified 14,447 SNPs were analyzed to estimate the LD patterns (r^2^) and LD decay extent across 11 chromosomes of mungbean. The LD patterns in a population of 127 AM panel genotypes showed that the LD decay was to be between the physical distances of 0–100 kb (around 57 kb) in mungbean chromosomes (Figure 5). The high resolution LD patterns resulting from a large number of SNP markers facilitate the higher mapping resolution in marker trait association analysis.

### Marker trait association (MTA) analysis

**FIGURE 6:**
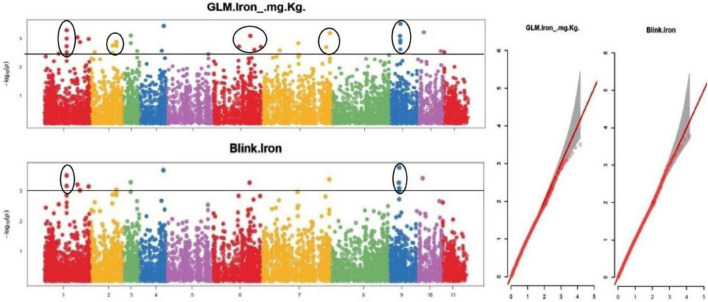
Manhattan plots and Quantile-Quantile plots depicting the significant association of SNP markers with grain iron concentration.

**FIGURE 7:**
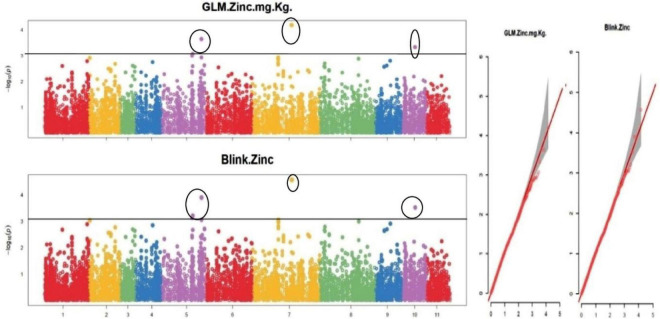
Manhattan plots and Quantile-Quantile plots depicting the significant association of SNP markers with grain zinc concentration.

**FIGURE 8:**
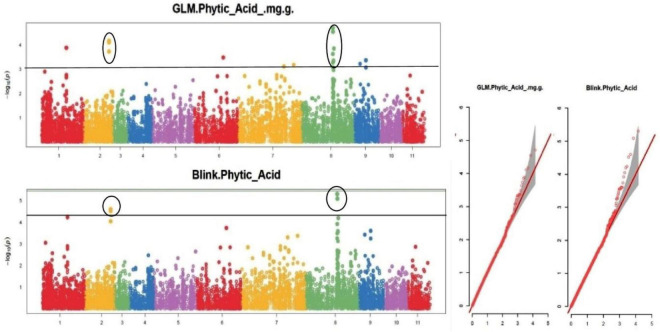
Manhattan plots and Quantile-Quantile plots depicting the significant association of SNP markers with grain phytic acid content.

**FIGURE 9:**
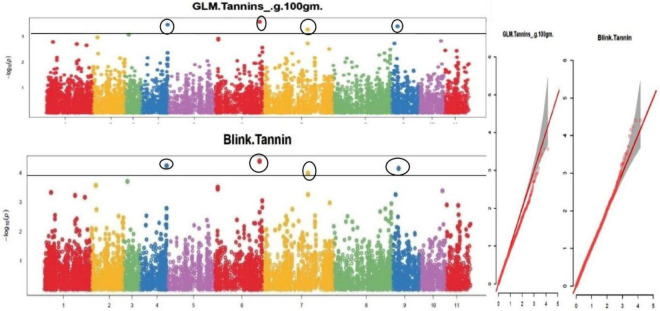
Manhattan plots and Quantile-Quantile plots depicting the significant association of SNP markers with grain tannin content.

The default GAPIT parameters were used, as well as a model selection with Bayesian Information Criterion (BIC), which determines the degree of population structure that should be accounted for in a model to avoid overfitting. Further, in this study, the Bonferroni correction threshold value of –log10 > 3.0 (*p*-value) was used as cut off to identify the significant SNPs associated with the grain iron and grain zinc concentration while –log10 > 4.0 (*p*-value) was used as cut off to identify the significant SNPs associated with for grain phytic acid and grain tannin content. The markers considered to be significantly associated with tested traits were represented by illustrating the Manhattan plots. Significant SNPs were identified from the BLINK model for (a) grain iron concentration (Figure 6) (b) grain zinc concentration (Figure 7) (c) grain phytic acid content (Figure 8) and (d) grain tannin content (Figure 9) across all chromosomes. The Fe showed strong association with SNPs present on chromosomes 1 and 9 exclusively. While, Zn exhibited significant linkage with SNPs present on chromosomes 5 and 7. In case of Phytic acid chromosome 8, having significant SNPs linked and Tannin content indicated strong association with SNPs found on chromosomes 6, 4, and 9. These SNPs were in local LD with multiple candidate genes. Summary table for studied traits and respective SNP were indicated in Table 2.

**TABLE 2:** Details of SNPs on different chromosomes and their corresponding putative genes associated with traits studied.

Sl. No.	Trait	Chromosome	No. of SNP	Blink model	GLM model			No. of putative candidate genes
				**Log_–10*P*_ range**	**(Log_–10*P*_) range**	**R square range**	**Phenotypic variation explained (%)**	
1	Grain iron	1	6	3.019–3.510	2.8674–3.2844	0.118–0.12656	11.8–12.6	18
2	Grain iron	2	1	3.038	2.883721	0.111	11.1	1
3	Grain iron	3	1	3.288	3.09	0.119	11.9	12
4	Grain iron	4	1	3.688	3.43155	0.132	13.2	1
5	Grain iron	6	1	3.273	3.08	0.122	12.2	5
6	Grain iron	7	1	3.380	3.17	0.122	12.2	11
7	Grain iron	9	3	3.090–3.7789	2.298–3.506	0.1131–0.13506	11.31–13.50	28
8	Grain iron	10	1	3.420	3.209	0.124	12.40	3
9	Grain Zn	2	1	3.033	2.910	0.102	10.20	12
10	Grain Zn	5	5	3.89–3.050	2.93–3.644	0.1025–0.1296	10.25–12.96	37
11	Grain Zn	7	3	3.050–4.568	2.929–4.186	0.1024–0.1515	10.24–15.15	1
12	Grain Zn	10	1	3.520	3.332	0.118	11.8	9
13	Grain PA	1	1	4.522	3.885	0.154	15.4	9
14	Grain PA	2	2	4.601	4.110–4.170	0.163–0.165	16.30–16.50	14
15	Grain PA	8	2	5.090–5.306	4.54–4.70	0.1805–0.1870	18.05–18.70	4
16	Grain TAN	4	1	4.243	3.451	0.120	12	6
17	Grain TAN	6	2	4.404	3.567	0.124	12.40	20
18	Grain TAN	7	1	4.001	3.265	0.113	11.30	1
19	Grain TAN	9	1	4.155	3.387	0.117	11.70	1

### Delineation of putative candidate genes

Total of 15 SNPs were found to be associated with the grain iron concentration. There were total 38 protein coding genes in the LD region of these SNPs. These genes were found to be involved in various protein formation, some of which are homeobox leucine zipper protein, WRKY family transcription factor, Pentatricopeptide repeat (PPR-like) superfamily protein, stress upregulated protein, Cytochrome P450 superfamily protein, iron ion binding and heme binding protein. Total of 10 SNPs were found to be associated with the grain zinc concentration and 59 genes were present in the haplotype of these SNPs. These were found to be associated with protein and enzyme formation like adenylate cyclase, zinc finger family protein, magnesium ion binding protein and protein kinase family protein. 5 SNPs which had 27 putative candidate genes in their haplotype were detected to be associated with grain phytic acid content (gene description are presented in Supplementary Table 10) which were found to be involved in several protein and enzyme formation with examples of Serine/threonine protein phosphatase family protein, Tubby like protein, Serine/Threonine kinase family protein and Phosphatidyl inositol kinase (PIK-G1)n. The study also found 4 SNPs having 26 genes in haplotype, associated with grain tannin content which were involved in synthesis of serine/threonine-protein phosphatase, triacylglycerol lipase, heat shock transcription factor B4, Sugar transporter SWEET n etc. proteins. *Vradi08g09870* was found to be significantly associated with grain phytic acid content, *Vradi04g09970* was found to be significantly associated with grain iron concentration, *Vradi07g13710* was found to be significantly associated with gain zinc concentration and *Vradi06g15120* was found to be significantly associated with grain tannin content (Supplementary Tables 8–11). Furthermore, among the putative candidate genes found for grain iron concentration three genes were found to be containing missense SNP in their CDS region. These missense SNPs were found to be involved in changing of the amino acids from C/T; valine to alanine, A/C; serine to tyrosine, G/T; serine to alanine, respectively, for genes *Vradi04g09970, Vradi06g11980*, and *Vradi09g05480*. Also there was one SNP in CDS region of the gene *Vradi07g30210*, but it caused a same sense mutation resulting in no structural change. So these genes may regulate the iron concentration in the mungbean grain by affecting the protein structure at tertiary level. Circular diagram depicting a summarized view of the significant association of SNP markers with all the four traits in the study along with SNP density in the outer ring (Supplementary Figure 3).

**FIGURE 10:**
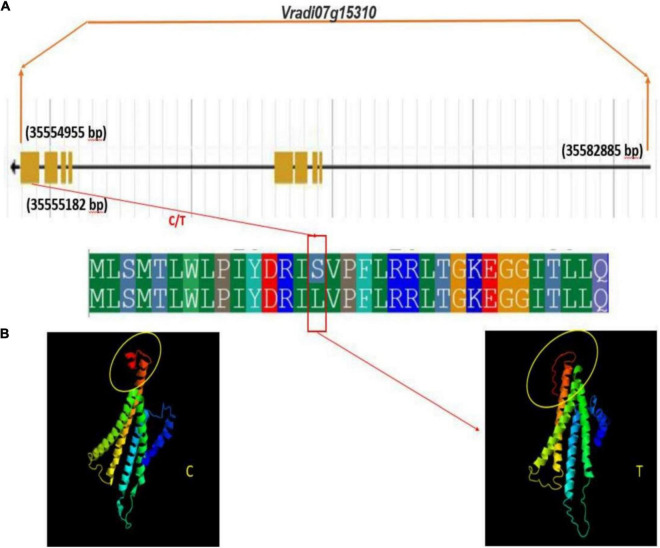
Gene and protein structure of potential candidate gene *Vradi07g15310* **(A)** gene structure **(B)** conformational changes in protein structure due to sense SNP (C/T) at the CDS domain.

Interestingly, a change in protein structure was observed for one of these genes *Vradi07g15310* by I-TASSER (see text footnote 5) (Figure 10). The structural analysis of these genes revealed a significant variation in native and mutated versions at protein level.

Therefore, it may be concluded that these are potential candidate genes involved in the regulation of tannin content in mungbean grains. These results showed that the identified SNPs and candidate genes are useful and worthy of being used in developing lines having low tannin content. In case of other traits, study did not observe any conformational changes with respect to CDS domain.

## Discussion

Mungbean is grown by resource-poor farmers since it only requires minimal irrigation and other inputs. It also replenishes soil fertility through symbiotic nitrogen fixation, is a drought-tolerant crop, and can survive high temperatures (average 35°C). Mungbean, being a substantial source of protein, is crucial for the country’s vegetarian population. It contains 25–31% of crude protein (51), 4–6 mg/100 g of iron (52), 355–375 Kcal/100 g of energy, and 1–5% crude fiber (53). Considering mungbean is a nutrient-dense legume with the potential to be mineral-dense, it really is a significant issue. Iron and zinc are vital minerals, and anemia caused by a lack of iron is a big issue. The results in NFHS 2019–21, the fifth in the series, show that across all age groups, children aged 6–59 months experienced the greatest increase in anemia, rising to 67.1% (NFHS-5) from 58.6% (NFHS-4, 2015–16). According to the information, the number was larger in rural India (68.3%) than in urban India (64.2%). Anemia affects 59.1% of females aged 15–19 years (NFHS-5), up from 54.1 percent in the previous year (NFHS-4). In this group as well, rural India had a higher percentage (58.7%) than urban India (54.1 per cent). 52.2% of pregnant women aged 15–49 years were found to be anemic, up from 50.4% in the previous survey. However, there is a significant gap between metropolitan areas (45.7%) and countryside India in this group (54.3%). According to the NFHS-5 data, 35.5% of kids under the age of five are stunted (height-for-age), compared to 38.8% in the NFHS-4. In 2007, a survey was done in Hisar-1 and Barwala block of Haryana state to determine the incidence of iron deficiency anemia and its relationship to dietary intake patterns of local communities (54). In these areas, 58% of the schoolchildren were anemic, with 49% of them lacking sufficient iron. The food quality in these areas was poor, with low iron bioavailability ranging between 3.1 and 4.6 percent, compared to healthy adult iron absorption of 10–15%.

Additionally, the discovery of QTLs/genes for grain iron and zinc concentrations, as well as grain phytic acid and grain tannin content features, allows for marker assisted selection to improve micronutrient content and bioavailability to consumers. The advancement of next-generation sequencing (NGS) technology in recent years has allowed for the effective characterization of genotypes at the molecular level (55). It also offers the greatest platform for studies such as genome-wide association mapping, which identifies SNP markers and candidate genes that are significantly related with attributes with high resolution (56).

The di-acid digestion method has been proven to be a reliable approach for determining micronutrients such as iron and zinc in organic samples (23). The Megazyme kit’s phytic acid estimation also delivers an accurate measurement of phytic acid content. A significant negative connection (*R*^2^ = −0.28) was identified between grain iron concentration and grain phytic acid content in this study. Akond et al. (57) found a similar trend in common bean. While there was absolutely no association between grain iron and grain zinc (*R*^2^ = 0.07), there was a loose positive link between Tannins and zinc (*R*^2^ = 0.06) and phytic acid (*R*^2^ = 0.07) (0.01). Furthermore, there was no significant relationship between zinc and grain phytic acid levels (*R*^2^ = −0.03). Despite the fact that (58) identified a substantial positive association between grain iron and grain zinc concentration, this investigation discovered a non-significant but positive correlation. This could be due to the diverse genotypes utilized in the study and the geographic positions where the experiments were conducted. The findings indicate that the accumulation and augmentation of one mineral has no effect on the concentration of others, and that they are inherited separately in the mungbean genome, which is consistent with Welch and Graham’s (59) findings. The findings of House et al. (60) in common bean further support the lack of a link between grain zinc concentration and grain phytic acid level. Furthermore, the levels of Fe, Zn, Phytic acid, and Tannin in this investigation were comparable to prior studies (61–64).

PUSA 1333 and IPM 02–19 genotypes were identified to exhibit high grain iron and zinc concentrations while having low grain phytic acid and tannin content. GANGA 8, IC 436637, KM 16–82, MH 1442, and TM 96–2 genotypes had high grain iron content, moderate grain zinc content, and low grain phytic acid and tannin content.

### The genetic diversity among mungbean genotypes using SNP markers

Molecular markers have been extensively utilized in the mungbean for molecular characterization, genetic diversity, and gene tagging (65). Molecular characterization of 127 different mungbean genotypes was performed in this study utilizing SNP markers. In other legumes like Chickpea (28), common bean (66), and in cereals like rice (67), wheat (68), and maize (69) have all employed SNP markers developed through GBS for diversity study. GBS approach is successfully used for the generation of SNP markers in mungbean (70) after the development of the reference genome (31). Noble et al. (71) and Breria et al. (72) used GBS technology to develop 22,230 and 24,870 SNP markers in mungbean, respectively. A total of 14,447 high-quality and non-erroneous SNPs were generated with comprehensive genome coverage in this study. As a result, SNPs with an average sequence read length of more than 150 bp were found in longer, high-quality sequence reads in our investigation. The maximum and minimum of SNPs were observed in ours study was in line with Noble et al. (71) and Breria et al. (72), on chromosome 1 (2,437 SNPs) and chromosome 3 (652 SNPs), respectively.

The population structure study with ADMIXTURE v 1.3.0 software revealed the presence of four subpopulations in analyzed 127 genotypes. Although the phylogenetic tree built by TASSEL v5.0 software utilizing neighbor end joining strategy revealed the presence of three subpopulations, one subpopulation obtained with this approach was particularly vast and was further separated into two groups. Noble et al. (71) and Breria et al. (72) previously reported the occurrence of four subpopulations among the 466 different mungbean accessions and 297 mungbean minicore collections, respectively. Versha et al. (73) recently discovered four subpopulations in an association mapping analysis comprising 80 genotypes. The genomic resources developed in this study will pave the way for the discovery of SNPs/candidate genes linked to agronomic traits in mungbean.

Researchers must first evaluate the degree of linkage disequilibrium (LD) and its degradation before conducting a genome-wide association mapping analysis in a population. A high resolution LD pattern in a population of 127 genotypes was observed in this study, with an LD estimate of 0.62 *r*^2^-value in a population of 127 genotypes. In mungbean chromosomes, the LD decline (decrease of *r*^2^-value to half of its highest) was seen between 0 and 100 kb (about 57.67 kb). LD degradation was seen at 60 and 100 kb physical distances for wild and cultivated mungbean genotypes, respectively, in a previous study (71). Furthermore, in a population of 297 mungbean minicore collections, Breria et al. (72) showed LD decline at a physical distance of 350 kb. The LD degradation seen in this study was likely similar to that observed in other legume crops such as soybean (74), but differed from that found in chickpea (28) at a physical distance of 1,000 kb.

The mineral bioavailability to the consumer is determined by complex features such as grain iron, zinc concentration, and grain phytic acid and tannin content. As a result, dissecting the genetic architecture of these quantitative features in crop plants is critical. For dissecting complex features in crop plants, the association mapping (AM) technique has evolved as a strong and alternative tool to biparental mapping. This method has been used to successfully identify markers/candidate genes associated with grain iron and grain zinc traits in a variety of crop plants, including chickpea (75), wheat (76, 77), pearlmillet (78), and the mungbean itself (58). Till date, just one study in mungbean has used the association mapping approach, and that study was conducted in the USDA core collection (58). For the first time in mungbean, a variety of Indian and exotic lines were used in an association mapping technique for grain iron and zinc.

Correspondingly, the association mapping strategy has been successful in identifying markers/candidate genes associated with grain phytic acid in a variety of crop plants, including *Brassica rapa* (79), rice (80), common bean (81), and most recently pea (82). However, association mapping has not been used to find markers linked with grain phytic acid levels in the mungbean crop. SNPs/Candidate genes linked with grain phytic acid content were found for the first time in mungbean using an association mapping approach in this study.

In numerous crop plants, including sorghum (83, 84) and rape seed (85), the association mapping approach has been successful in identifying the markers/candidate genes linked with grain tannin concentration. However, association mapping has not been used to find markers linked with grain tannin content in the mungbean crop. SNPs/Candidate genes linked with grain tannin concentration in mungbean were identified for the first time in this study using an association mapping approach.

To create the AM panel for this investigation, 127 different mungbean genotypes were selected and genotyped using 14,447 SNPs. The general linear model (GLM) and Bayesian-information and Linkage-disequilibrium Iteratively Nested Keyway (Blink) techniques were used to analyze the associations. Furthermore, a cut-off value of –log10 > 3.0 was used to identify significant SNPs linked to grain iron and zinc concentrations, while a cut-off value of –log10 > 4.0 was used to identify significant SNPs linked to grain phytic acid and tannin content. The GLM found 9, 6, 5, and 0 SNPs linked to grain iron concentration, zinc concentration, phytic acid content, and tannin content, respectively. While Blink identified 15, 10, 5, and 5 SNPs linked with grain iron, grain zinc, grain phytic acid content, and grain tannin content, correspondingly.

The multigenic regulation of nutrient accumulation in mungbean seeds found in this work coincides with (86) findings of quantitative inheritance. In a mung bean RIL population, these researchers discovered 17 QTLs for Fe and Zn, including seven QTLs on linkage groups LG 6 and LG 7 for Zn and one QTL shared with Fe. In this study, LG 6 and LG 7 each had one QTL for iron, but LG7 had three QTLs for zinc. On the LG 11 map, Singh (87) discovered a potential QTL (qFe-11-1) for iron. One QTL for iron was found at LG11 in this study. In 2020, Wu et al. discovered SNPs associated with grain iron content on LG 06, and an SNP was discovered on LG 06 in this study as well. Wu et al. (58) discovered SNPs linked with grain zinc content on LG 07 in 2020, and three SNPs were located on LG 07 in this study as well.

The 35 SNPs detected by Blink were shown to be linked to 170 protein-coding genes and 11 unidentified genes. Iron ion/heme binding proteins, Glutathione-S transferase (GST), major intrinsic (MIP) protein family, WRKY family transcription factors, squamosal promoter binding proteins, and ATP dependent metalloproteases are among the proteins coding genes for grain iron content. In flowering plants, the WRKY gene family encodes a vast number of transcription factors (TFs) that are involved in a variety of root development, stress responses, developmental, and physiological activities (88). Plant-specific transcription factors encoded by Squamosa Promoter-Binding Protein-Like (SPL) genes serve critical roles in plant phase transition, flower and fruit development, and plant architecture (89). Aquaporin proteins, which are members of the big major intrinsic (MIP) protein family, are the primary facilitators of water transport activity through plant cell membranes. These proteins appear to govern the transcellular route of water (90) and play a critical role in delivering a high volume of water with minimal energy expenditure (91). A variety of stress-response genes are up-regulated in an ATP-dependent metalloprotease with a high level of reactive oxygen species (ROS) (92). Glutathione-S transferases (GST) have been used in a variety of plant functions, including xenobiotic detoxification, secondary metabolism, growth and development, and, most importantly, protection against biotic and abiotic stimuli (93).

Adenylate cyclase, Pentatricopeptide repeat (PPR) superfamily protein, zinc finger (Ran-binding) family protein, magnesium ion binding protein, copper ion binding protein, major intrinsic protein (MIP) family transporter, and protein kinase family protein are the most important protein coding genes for grain zinc concentration.

Protein coding genes for grain phytic acid content consists of Serine/threonine protein phosphatase family protein, serine/threonine kinase, transmembrane amino acid transporter family protein, callose synthase, Phosphatidyl inositol kinase (PIK-G1) n, and Cytochrome P450 superfamily protein. Serine/threonine protein phosphatase family protein plays a prominent role in the regulation of specific signal transduction cascades, as witnessed by its presence in a number of macromolecular signaling modules, where it is often found in association with other phosphatases and kinases (94). The network of protein serine/threonine kinases in plant cells act as a “central processor unit” (cpu), accepting input information from receptors that sense environmental conditions, phytohormones, and other external factors, and converting it into appropriate outputs such as changes in metabolism, gene expression, and cell growth and division (95). According to Lee et al. (96), phosphatidylinositol 3-kinase is essential for vacuole reorganization and nuclear division during pollen development. Phosphatidylinositol 3-phosphate (PtdInsP) is made by the enzyme phosphatidylinositol 3-kinase (PI3K), which phosphorylates phosphoinositides at the D-3 position. PtdIns(3)P is required for normal plant growth (97) and has been linked to a number of physiological processes, including root nodule formation (98), auxin-induced production of reactive oxygen species and root gravitropism (99), root hair curling and Rhizobium infection in *Medicago truncatula* (100), increased plasma membrane endocytosis and the intracellular production of reactive oxygen species in salt tolerance response (101), stomatal closing movement (102, 103), and root hair elongation (100). The cytochrome P450 (CYP) superfamily is the largest enzymatic protein family in plants. Members of this superfamily are involved in multiple metabolic pathways with distinct and complex functions, playing important roles in a vast array of reactions. As a result, numerous secondary metabolites are synthesized that function as growth and developmental signals or protect plants from various biotic and abiotic stresses (104).

While the protein coding genes for grain tannin content consists of serine/threonine-protein phosphatase, heat shock transcription factor B4, triacylglycerol lipase, serine/threonine kinase, Sugar transporter SWEET n, and Nitrate transporter. As discussed earlier the serine/threonine-protein phosphatase plays a prominent role in the regulation of specific signal transduction cascades and control the changes in metabolism, gene expression, and cell growth and division (95). The enhanced heat shock gene expression in response to various stimuli is regulated by heat shock transcription factors (HSFs) (105) which may have some correlation to the tannin content in grain considering tannins are related to stress response. SWEET (Sugars Will Eventually Exported Transporters) proteins are one of the biggest sugar transporter families in the plant kingdom, and they play an important role in plant growth and stress responses (106). SWEET genes’ various functions in critical developmental and physiological processes including as growth, senescence, and flower/seed/pollen formation are similarly explained in higher plants. They are also known to have a role in abiotic and biotic stress adaptation, as well as host-pathogen interactions (107–113). This gives some hints on the relation of the SWEET gene and the tannin content in the mungbean grain. The inclusion of nitrogen transporters in the list of associated genes with tannins suggests possibility of some relation between nitrogen assimilation and tannin content in mungbean grain.

There were 11 uncharacterized genes detected in relation with all of the attributes studied, necessitating more research to determine their function and potential impact on the phenotypic of the trait in question in mungbean grain.

Among these putative candidate genes, genes namely *Vradi04g09970, Vradi07g30210, Vradi06g11980, Vradi09g05480*, and *Vradi07g15310* were identified with missense SNPs in their CDS region. Further, structural changes at protein level due to missense SNPs in their CDS region were observed for two genes namely *Vradi07g15310 and Vradi09g05480.* The allelic variation between native and mutant versions of these genes reveals the discrepancy in protein structure and domains for modification at post transcriptional level. The variation in protein-protein interactions or signal integration leads to a difference in transcriptional modulations that result in an observed phenotypic difference in grain iron concentration and tannin content.

After Wu et al. (58), the current study is the first to report on association mapping of grain phytic acid and tannin content in mungbean, as well as the second to report on association mapping of grain iron and grain zinc concentration in mungbean, however, this study looked at different genotypes. In mungbean breeding programs focusing on bio-fortification and increased nutritional availability, the found SNP markers and candidate genes are useful resources. Furthermore, this research demonstrates that association mapping, particularly using the Blink model, is a powerful tool for dissecting complex traits such as grain iron concentration, grain zinc concentration, grain phytic acid content, and grain tannin content, and provides high resolution mapping at a low cost and in a short amount of time.

## Conclusion

• The genotypes PUSA 1333 and IPM 02–19 were identified as desired genotypes as they had high grain iron and zinc concentration but low grain phytic acid and tannin contents.
• The study generated 14,447 genome wide SNPs by employing next generation sequencing (NGS) based genotyping by sequencing (GBS) methodology.
• Population admixture analysis revealed the presence of four different ancestry among the 127 genotypes and LD decay of ∼57.6 kb physical distance was observed in mungbean chromosomes.
• Association mapping analysis revealed that a total of 20 significant SNPs were shared by both GLM and Blink models associated with grain micronutrient and anti-nutritional factor traits.
• The study identified the 185 putative candidate genes including potential candidate genes *Vradi07g30190*, *Vradi01g09630*, and *Vradi09g05450* were found to be associated with grain iron concentration, *Vradi10g04830* with grain zinc concentration, *Vradi08g09870* and *Vradi01g11110* with grain phytic acid content and *Vradi04g11580* and *Vradi06g15090* with grain tannin content.
• Two genes *Vradi07g15310 and Vradi09g05480* showed significant variation in protein structure between native and mutated versions. The identified SNPs and candidate genes are potential powerful tools for nutritional improvement in mungbean breeding program.

## Data availability statement

The original contributions presented in this study are included in the article/Supplementary material, further inquiries can be directed to the corresponding authors.

## Author contributions

MA, GM, and HD: conceptualization and supervision. MS, JT, PY, MK, MA, and RN: methodology. MK, MA, HD, and AP: formal analysis. RN, SK, RS, and HD: resources. MS and MA: data curation. MS, MA, and RN: writing—original draft preparation. MA, MS, RS, RN, AP, and SG: writing—review and editing. All authors contributed to the article and approved the submitted version.

## Footnotes

1
http://ods.od.nih.gov/factsheets/zinc-HealthProfessional/

2
http://creskolab.uoregon.edu/stacks

3
https://legacy.legumeinfo.org/genomes/jbrowse/?data=Vr1.0

4
https://web.expasy.org/translate/

5
https://zhanggroup.org/I-TASSER/

## Supplementary material

Supplementary Figure 1 Determination of Q matrix with the lowest cross-validation error (in this case *k* = 4) (X-axis has the values of *k* while the Y-axis contains the values of CV at corresponding *k*).

Supplementary Figure 2 Phylogenetic tree depicting the genetic relations among 127 diverse mungbean genotypes based on Nei’s genetic distance using 14,447 high quality GBS based SNPs.

Supplementary Figure 3 Circular diagram depicting a summarized view of the significant association of SNP markers with all the four traits in the study along with SNP density in the outer ring.
