# Supplementary material for: Genome wide association analysis for grain micronutrients and anti-nutritional traits in mungbean [Vigna radiata (L.) R. Wilczek] using SNP markers
Source: Front Nutr. 2023 Feb 7;10:1099004. doi: 10.3389/fnut.2023.1099004 (PMC9941709; doi:10.3389/fnut.2023.1099004)
Supplement: Supplementary file 1 [file Data_Sheet_1.docx]

**Supplementary Table 1. List of mungbean genotypes used in the study**

| **Sl. No.** | **Names** | **Source/origin** | **Sl. No.** | **Names** | **Source/origin** |
| --- | --- | --- | --- | --- | --- |
| **1** | BASANTI | CCSHAU, India | **77** | M 837 | AVRDC, Taiwan |
| **2** | Bhutan LM 1 | Bhutan | **78** | M 875 | AVRDC, Taiwan |
| **3** | Bhutan LM 2 | Bhutan | **79** | M 880 | IARI, India |
| **4** | Bhutan LM 95 | Bhutan | **80** | M 906 | AVRDC, Taiwan |
| **5** | China Mung | China | **81** | M 958 | AVRDC, Taiwan |
| **6** | DMS10 | Bihar, India | **82** | M 961 | AVRDC, Taiwan |
| **7** | DMS 8 | Bihar, India | **83** | M 981 | AVRDC, Taiwan |
| **8** | DMS-4 | Bihar, India | **84** | M 989 | AVRDC, Taiwan |
| **9** | EC 3988891 | AVRDC, Thailand | **85** | M1129 | AVRDC, Taiwan |
| **10** | EC 520024 | AVRDC, Thailand | **86** | M1209 | AVRDC, Taiwan |
| **11** | EC 520029 | AVRDC, Thailand | **87** | M1316 | AVRDC, Taiwan |
| **12** | EC 520041 | AVRDC, Thailand | **88** | M1334 | AVRDC, Taiwan |
| **13** | EC 550851 | AVRDC, Thailand | **89** | M1350 | AVRDC, Taiwan |
| **14** | GANGA 1 | Rajasthan, India | **90** | M1354 | AVRDC, Taiwan |
| **15** | GANGA 8 | Rajasthan, India | **91** | M1429 | AVRDC, Taiwan |
| **16** | HUM 1 | BHU, India | **92** | M1443 | PAU, India |
| **17** | HUM 16 | BHU, India | **93** | M186 | Bihar, India |
| **18** | HUM 2 | BHU, India | **94** | M322 | IARI, India |
| **19** | HUM 6 | BHU, India | **95** | M409 | - |
| **20** | IC 28083 | NBPGR, India | **96** | M450 | IARI, India |
| **21** | IC 282094 | NBPGR, India | **97** | M660 | PAU, India |
| **22** | IC 325828 | NBPGR, India | **98** | M-704 | - |
| **23** | IC 436637 | NBPGR, India | **99** | M729 | Philippines |
| **24** | IC 436763 | NBPGR, India | **100** | M739 | Philippines |
| **25** | IC 546476 | NBPGR, India | **101** | MH 1442 | CCSHAU, India |
| **26** | IC 546488 | NBPGR, India | **102** | MH 215 | CCSHAU, India |
| **27** | IC436636 | NBPGR, India | **103** | MH 318 | CCSHAU, India |
| **28** | IPM 02-14 | IIPR, India | **104** | MH 565 | CCSHAU, India |
| **29** | IPM 02-15 | IIPR, India | **105** | MH 810 | CCSHAU, India |
| **30** | IPM 02-17 | IIPR, India | **106** | MH 934 | CCSHAU, India |
| **31** | IPM 02-19 | IIPR, India | **107** | MH 96-1 | CCSHAU, India |
| **32** | IPM 02-3 | IIPR, India | **108** | ML 1299 | PAU, India |
| **33** | IPM 02-30 | IIPR, India | **109** | ML 1451 | PAU, India |
| **34** | IPM 205-4 | IIPR, India | **110** | ML 1464 | PAU, India |
| **35** | IPM 205-7 | IIPR, India | **111** | ML 1628 | PAU, India |
| **36** | IPM 288 | IIPR, India | **112** | ML 2037 | PAU, India |
| **37** | IPM 406-1 | IIPR, India | **113** | ML 818 | PAU, India |
| **38** | IPM 409-4 | IIPR, India | **114** | MUSKAN | CCSHAU, India |
| **39** | IPM 410-3 | IIPR, India | **115** | NM 1 | NDUAT, India |
| **40** | KM 11-40 | AICRP, MULLaRP, India | **116** | OLRM 4 | Orissa, India |
| **41** | KM 12-28 | AICRP, MULLaRP, India | **117** | PDM 139 | IIPR, India |
| **42** | KM 16-23 | AICRP, MULLaRP, India | **118** | PLM 167 | AVRDC, Thailand |
| **43** | KM 16-60 | AICRP, MULLaRP, India | **119** | PLM 271 | AVRDC, Thailand |
| **44** | KM 16-69 | AICRP, MULLaRP, India | **120** | PrakashNepal | Nepal |
| **45** | KM 16-75 | AICRP, MULLaRP, India | **121** | PS 16 | IARI, India |
| **46** | KM 16-80 | AICRP, MULLaRP, India | **122** | PUSA 0971 | IARI, India |
| **47** | KM 16-82 | AICRP, MULLaRP, India | **123** | PUSA 1131 | IARI, India |
| **48** | KM 2241 | AICRP, MULLaRP, India | **124** | PUSA 1132 | IARI, India |
| **49** | KM 7-134 | AICRP, MULLaRP, India | **125** | PUSA 1331 | IARI, India |
| **50** | KM16-18 | AICRP, MULLaRP, India | **126** | PUSA 1332 | IARI, India |
| **51** | LGG 460 | ANGRAU, India | **127** | PUSA 1333 | IARI, India |
| **52** | M 1032 | AVRDC, Taiwan | **128** | PUSA 1341 | IARI, India |
| **53** | M 1053 | ANGRAU, India | **129** | PUSA 1342 | IARI, India |
| **54** | M 1131 | AVRDC, Taiwan | **130** | PUSA 1441 | IARI, India |
| **55** | M 1156 | AVRDC, Taiwan | **131** | Pusa Ratna | IARI, India |
| **56** | M 1168 | AVRDC, Taiwan | **132** | Pusa Vishal | IARI, India |
| **57** | M 1255 | AVRDC, Taiwan | **133** | Pusa0871 | IARI, India |
| **58** | M 1370 | AVRDC, Taiwan | **134** | Pusa9531 | IARI, India |
| **59** | M 1372 | AVRDC, Taiwan | **135** | PusaBaisakhi | IARI, India |
| **60** | M 1378 | AVRDC, Taiwan | **136** | RMG 1028 | Rajasthan, India |
| **61** | M 1400 | AVRDC, Taiwan | **137** | RMG 1087 | Rajasthan, India |
| **62** | M 1447 | IIPR, India | **138** | RMG 991 | Rajasthan, India |
| **63** | M 145 | IARI, India | **139** | RMGP 1 | Rajasthan, India |
| **64** | M 1477 | Uttar Pradesh, India | **140** | SATYA | CCSHAU, India |
| **65** | M 1485 | Maharastra, India | **141** | TM 96-2 | BARC, India |
| **66** | M 1493 | Pantnagar, India | **142** | TM 96-25 | BARC, India |
| **67** | M 1503 | ANGRAU, India | **143** | TM 9725 | BARC, India |
| **68** | M 204 | Bihar, India | **144** | V 04-04 | AVRDC, Thailand |
| **69** | M 289 | IARI, India | **145** | V 1109 | AVRDC, Thailand |
| **70** | M 313 | IARI, India | **146** | V 1138 | AVRDC, Thailand |
| **71** | M 422 | IARI, India | **147** | V 1153 | AVRDC, Thailand |
| **72** | M 499 | Uttar Pradesh, | **148** | V 3518 | AVRDC, Thailand |
| **73** | M 678 | Hisar, India | **149** | V 6183 | AVRDC, Thailand |
| **74** | M 684 | Hisar, India | **150** | Yellow Mung 1 | BCKV, India |
| **75** | M 700 | IARI, India |  |  |  |
| **76** | M 765 | IARI, India |  |  |  |

**Supplementary Table2. List of 127 mungbean association mapping panel genotypes along with their accession IDs submitted to NCBI-sequence read archive (SRA) database**

| S. | Genotype | Accession ID | Type | S. | Genotype | Accession ID | Type |
| --- | --- | --- | --- | --- | --- | --- | --- |
| No |  |  |  | No |  |  |  |
| 1 | BASANTI | SRR11210847 | RV | 64 | M 204 | SRR11210821 | GL |
| 2 | Bhutan LM 1 | SRR11210739 | RV | 65 | M 289 | SRR11210820 | GL |
| 3 | Bhutan LM 2 | SRR11210728 | RV | 66 | M 313 | SRR11210819 | GL |
| 4 | Bhutan LM 95 | SRR11210717 | RV | 67 | M 422 | SRR11210818 | GL |
| 5 | China Mung | SRR11210706 | RV | 68 | M 499 | SRR11210817 | GL |
| 6 | DMS 8 | SRR11210824 | ABL | 69 | M 678 | SRR11210815 | GL |
| 7 | EC 3988891 | SRR11210813 | GL | 70 | M 684 | SRR11210814 | GL |
| 8 | EC 520024 | SRR11210773 | GL | 71 | M 700 | SRR11210784 | GL |
| 9 | EC 520029 | SRR11210803 | GL | 72 | M 765 | SRR11210780 | GL |
| 10 | EC 520041 | SRR11210792 | GL | 73 | M 837 | SRR11210778 | GL |
| 11 | EC 550851 | SRR11210760 | GL | 74 | M 875 | SRR11210777 | GL |
| 12 | GANGA 1 | SRR11210749 | RV | 75 | M 880 | SRR11210776 | GL |
| 13 | GANGA 8 | SRR11210744 | RV | 76 | M 906 | SRR11210775 | GL |
| 14 | HUM 1 | SRR11210743 | RV | 77 | M 958 | SRR11210774 | GL |
| 15 | HUM 16 | SRR11210742 | RV | 78 | M 981 | SRR11210772 | GL |
| 16 | HUM 2 | SRR11210741 | RV | 79 | MH 1442 | SRR11210771 | ABL |
| 17 | HUM 6 | SRR11210740 | RV | 80 | MH 215 | SRR11210770 | RV |
| 18 | IC 28083 | SRR11210738 | GL | 81 | MH 318 | SRR11210769 | RV |
| 19 | IC 282094 | SRR11210737 | GL | 82 | MH 565 | SRR11210768 | ABL |
| 20 | IC 325828 | SRR11210735 | GL | 83 | MH 810 | SRR11210767 | RV |
| 21 | IC 436637 | SRR11210734 | GL | 84 | MH 934 | SRR11210766 | ABL |
| 22 | IC 436763 | SRR11210733 | GL | 85 | MH 96-1 | SRR11210765 | RV |
| 23 | IC 546476 | SRR11210732 | GL | 86 | ML 1299 | SRR11210764 | GL |
| 24 | IC 546488 | SRR11210731 | GL | 87 | ML 1451 | SRR11210763 | GL |
| 25 | IPM 02-19 | SRR11210730 | ABL | 88 | ML 1464 | SRR11210844 | GL |
| 26 | IPM 406-1 | SRR11210729 | ABL | 89 | ML 1628 | SRR11210812 | ABL |
| 27 | IPM 409-4 | SRR11210727 | ABL | 90 | ML 2037 | SRR11210811 | GL |
| 28 | IPM 410-3 | SRR11210726 | RV | 91 | ML 818 | SRR11210810 | RV |
| 29 | IPM 02-14 | SRR11210725 | RV | 92 | MUSKAN | SRR11210809 | RV |
| 30 | IPM 02-15 | SRR11210724 | ABL | 93 | NM 1 | SRR11210808 | RV |
| 31 | IPM 02-17 | SRR11210723 | ABL | 94 | OLRM 4 | SRR11210806 | GL |
| 32 | IPM 02-3 | SRR11210722 | RV | 95 | PDM 139 | SRR11210805 | RV |
| 33 | IPM 02-30 | SRR11210721 | ABL | 96 | PLM 167 | SRR11210804 | GL |
| 34 | IPM 205-4 | SRR11210720 | ABL | 97 | PLM 271 | SRR11210802 | GL |
| 35 | IPM 205-7 | SRR11210719 | RV | 98 | Prakash Nepal | SRR11210801 | RV |
| 36 | IPM 288 | SRR11210718 | ABL | 99 | PS 16 | SRR11210800 | RV |
| 37 | KM 11-40 | SRR11210716 | ABL | 100 | PUSA 0971 | SRR11210799 | RV |
| 38 | KM 12-28 | SRR11210715 | ABL | 101 | PUSA 1131 | SRR11210797 | ABL |
| 39 | KM 16-23 | SRR11210714 | ABL | 102 | PUSA 1132 | SRR11210796 | ABL |
| 40 | KM 16-60 | SRR11210712 | ABL | 103 | PUSA 1331 | SRR11210795 | ABL |
| 41 | KM 16-69 | SRR11210711 | ABL | 104 | PUSA 1332 | SRR11210794 | ABL |
| 42 | KM 16-75 | SRR11210710 | ABL | 105 | PUSA 1333 | SRR11210793 | ABL |
| 43 | KM 16-80 | SRR11210708 | ABL | 106 | PUSA 1341 | SRR11210791 | ABL |
| 44 | KM 16-82 | SRR11210705 | ABL | 107 | PUSA 1342 | SRR11210790 | ABL |
| 45 | KM 2241 | SRR11210845 | ABL | 108 | PUSA 1441 | SRR11210789 | ABL |
| 46 | KM 7-134 | SRR11210843 | ABL | 109 | PUSA 871 | SRR11210788 | ABL |
| 47 | LGG 460 | SRR11210842 | RV | 110 | Pusa Baisakhi | SRR11210787 | RV |
| 48 | M 1032 | SRR11210841 | GL | 111 | Pusa Ratna | SRR11210786 | RV |
| 49 | M 1053 | SRR11210840 | GL | 112 | Pusa Vishal | SRR11210785 | RV |
| 50 | M 1131 | SRR11210839 | GL | 113 | RMG 1028 | SRR11210783 | RV |
| 51 | M 1156 | SRR11210838 | GL | 114 | RMG 1087 | SRR11210762 | RV |
| 52 | M 1168 | SRR11210837 | GL | 115 | RMG 991 | SRR11210761 | RV |
| 53 | M 1255 | SRR11210836 | GL | 116 | RMGP 1 | SRR11210759 | RV |
| 54 | M 1370 | SRR11210833 | GL | 117 | SATYA | SRR11210758 | RV |
| 55 | M 1372 | SRR11210832 | GL | 118 | TM 96-2 | SRR11210756 | RV |
| 56 | M 1378 | SRR11210831 | GL | 119 | TM 96-25 | SRR11210755 | RV |
| 57 | M 1400 | SRR11210830 | GL | 120 | TM 9725 | SRR11210754 | ABL |
| 58 | M 1447 | SRR11210828 | GL | 121 | V 04-04 | SRR11210753 | GL |
| 59 | M 145 | SRR11210827 | GL | 122 | V 1109 | SRR11210752 | ABL |
| 60 | M 1477 | SRR11210826 | GL | 123 | V 1138 | SRR11210751 | ABL |
| 61 | M 1485 | SRR11210825 | GL | 124 | V 1153 | SRR11210750 | ABL |
| 62 | M 1493 | SRR11210823 | GL | 125 | V 3518 | SRR11210748 | ABL |
| 63 | M 1503 | SRR11210822 | GL | 126 | V 6183 | SRR11210747 | ABL |
|  |  |  |  | 127 | Yellow Mung 1 | SRR11210746 | GL |
| RV, released variety; ABL, advanced breeding line; GL, germplasm line | | | | | | | |

**Supplementary Table 3. ANOVA for augmented block design for nutritional and anti-nutritional traits**

| **Iron** | **df** | **Sum Sq** | **MSS** | **F value** | **Pr(>F)** |  |
| --- | --- | --- | --- | --- | --- | --- |
| **Block unadj** | 4 | 1865 | 466.20 |  |  |  |
| **Trt.adj** | 149 | 39135 | 262.65 | 20.134 | 1.502e-08 | *** |
| **Control** | 4 | 976 | 244.10 | 18.712 | 7.027e-06 | *** |
| **Control + control.VS.aug.** | 145 | 38159 | 263.16 | 20.174 | 1.493e-08 | *** |
| **Residual** | 16 | 209 | 13.04 |  |  |  |

| **Zinc** | **df** | **Sum Sq** | **MSS** | **F value** |  |  |
| --- | --- | --- | --- | --- | --- | --- |
| **Block unadj** | 4 | 114.7 | 28.67 |  |  |  |
| **Trt.adj** | 149 | 24825.5 | 166.61 | 27.774 | 1.275e-09 | *** |
| **Control** | 4 | 2194.6 | 548.66 | 91.459 | 8.236e-11 | *** |
| **Control + control.VS.aug.** | 145 | 22630.9 | 156.07 | 26.017 | 2.128e-09 | *** |
| **Residual** | 16 | 96.0 | 6.00 |  |  |  |

| **Phytic Acid** | **df** | **Sum Sq** | **MSS** | **F value** |  |  |
| --- | --- | --- | --- | --- | --- | --- |
| **Block unadj** | 4 | 13.59 | 3.396 |  |  |  |
| **Trt.adj** | 149 | 841.59 | 5.648 | 10.4647 | 1.975e-06 | *** |
| **Control** | 4 | 163.72 | 40.930 | 75.8325 | 3.416e-10 | *** |
| **Control + control.VS.aug.** | 145 | 677.87 | 4.675 | 8.6614 | 7.751e-06 | *** |
| **Residual** | 16 | 8.64 | 0.540 |  |  |  |

| **Tannin** | **df** | **Sum Sq** | **MSS** | **F value** |  |  |
| --- | --- | --- | --- | --- | --- | --- |
| **Block unadj** | 4 | 5.818 | 1.4546 |  |  |  |
| **Trt.adj** | 149 | 171.699 | 1.1523 | 128.03 | 7.801e-15 | *** |
| **Control** | 4 | 34.285 | 8.5713 | 952.28 | < 2.2e-16 | *** |
| **Control +control.VS.aug.** | 145 | 137.414 | 0.9477 | 105.29 | 3.708e-14 | *** |
| **Residual** | 16 | 0.144 | 0.0090 |  |  |  |

**Supplementary Table 4. Descriptive statistics for the investigated traits**

| **Iron (mg/Kg)** | | **Zinc(mg/Kg)** | | **Phytic Acid (mg/g)** | | **Tannins (g/100g)** | |
| --- | --- | --- | --- | --- | --- | --- | --- |
| Mean | 74.15 | Mean | 32.20 | Mean | 7.35 | Mean | 3.80 |
| Standard Error | 1.30 | Standard Error | 0.79 | Standard Error | 0.17 | Standard Error | 0.08 |
| Median | 72.35 | Median | 32.6 | Median | 7.094 | Median | 3.66 |
| Mode | 56.4 | Mode | 35.75 | Mode | 7.15 | Mode | 3.55 |
| Standard Deviation | 15.74 | Standard Deviation | 9.63 | Standard Deviation | 2.11 | Standard Deviation | 0.99 |
| Sample Variance | 247.93 | Sample Variance | 92.78 | Sample Variance | 4.48 | Sample Variance | 0.98 |
| Range | 73.65 | Range | 52.45 | Range | 13.35 | Range | 4.11 |
| Minimum | 48.2 | Minimum | 8.6 | Minimum | 1.5 | Minimum | 2.14 |
| Maximum | 121.85 | Maximum | 61.05 | Maximum | 14.85 | Maximum | 6.25 |
| CV | 21.23 | COV^a^ | 29.90 | COV^a^ | 28.80 | COV^a^ | 26.00 |

**Supplementary Table 5. Mean values of 145 mungbean genotypes for (a) grain iron concentration (b) grain zinc concentration (c) grain phytic acid content and (d) grain tannin content**

| **Genotypes** | **Names** | **Iron (mg/Kg)** | **Zinc(mg/Kg)** | **Phytic Acid (mg/g)** | **Tannins (g/100g)** |
| --- | --- | --- | --- | --- | --- |
| 1 | **BASANTI** | 81.40 | 61.05 | 4.79 | 5.06 |
| 2 | **Bhutan LM 1** | 58.40 | 27.90 | 7.13 | 3.81 |
| 3 | **Bhutan LM 2** | 69.35 | 32.90 | 6.06 | 3.33 |
| 4 | **Bhutan LM 95** | 63.40 | 29.80 | 8.59 | 3.73 |
| 5 | **China Mung** | 77.35 | 33.70 | 5.40 | 4.24 |
| 6 | **DM510** | 64.40 | 10.80 | 6.80 | 4.46 |
| 7 | **DMS 8** | 96.05 | 29.95 | 7.15 | 2.36 |
| 8 | **DMS-4** | 73.35 | 35.25 | 6.51 | 3.95 |
| 9 | **EC 3988891** | 54.40 | 26.50 | 6.65 | 3.75 |
| 10 | **EC 520024** | 71.35 | 25.25 | 6.02 | 5.08 |
| 11 | **EC 520029** | 56.40 | 19.60 | 13.80 | 3.26 |
| 12 | **EC 520041** | 56.40 | 10.30 | 8.50 | 3.41 |
| 13 | **EC 550851** | 80.35 | 36.70 | 6.80 | 3.34 |
| 14 | **GANGA 1** | 78.35 | 42.65 | 6.76 | 4.00 |
| 15 | **GANGA 8** | 121.85 | 33.65 | 3.00 | 2.67 |
| 16 | **HUM 1** | 60.40 | 36.40 | 10.13 | 3.56 |
| 17 | **HUM 16** | 74.35 | 47.50 | 6.33 | 3.56 |
| 18 | **HUM 2** | 85.30 | 34.00 | 7.13 | 2.84 |
| 19 | **HUM 6** | 65.40 | 35.10 | 7.76 | 3.48 |
| 20 | **IC 28083** | 75.95 | 41.40 | 4.99 | 6.25 |
| 21 | **IC 282094** | 81.30 | 30.55 | 6.94 | 5.34 |
| 22 | **IC 325828** | 70.35 | 59.45 | 9.06 | 3.25 |
| 23 | **IC 436637** | 97.00 | 35.10 | 4.70 | 2.81 |
| 24 | **IC 436763** | 85.30 | 29.45 | 14.36 | 4.26 |
| 25 | **IC 546476** | 80.75 | 35.75 | 6.74 | 5.24 |
| 26 | **IC 546488** | 64.40 | 36.55 | 7.21 | 5.53 |
| 27 | **IC436636** | 69.35 | 42.20 | 9.04 | 2.89 |
| 28 | **IPM 02-14** | 106.55 | 22.90 | 4.40 | 2.67 |
| 29 | IPM 02-15 | 67.35 | 38.90 | 9.10 | 6.23 |
| 30 | **IPM 02-17** | 67.35 | 32.45 | 7.83 | 4.13 |
| 31 | **IPM 02-19** | 104.65 | 46.40 | 1.50 | 2.89 |
| 32 | **IPM 02-3** | 52.25 | 13.30 | 6.84 | 4.23 |
| 33 | **IPM 02-30** | 61.40 | 37.95 | 8.73 | 4.51 |
| 34 | **IPM 205-4** | 81.70 | 28.35 | 7.29 | 5.50 |
| 35 | **IPM 205-7** | 92.30 | 26.30 | 8.09 | 3.09 |
| 36 | **IPM 288** | 68.35 | 26.95 | 5.90 | 2.14 |
| 37 | **IPM 406-1** | 88.40 | 33.25 | 5.38 | 4.58 |
| 38 | **IPM 409-4** | 97.25 | 28.20 | 6.24 | 2.97 |
| 39 | **IPM 410-3** | 100.35 | 25.25 | 6.49 | 5.29 |
| 40 | **KM 11-40** | 67.35 | 39.85 | 10.00 | 3.13 |
| 41 | **KM 12-28** | 57.40 | 35.75 | 7.23 | 4.26 |
| 42 | **KM 16-23** | 79.35 | 41.85 | 6.66 | 3.78 |
| 43 | **KM 16-60** | 78.35 | 26.15 | 7.54 | 3.23 |
| 44 | **KM 16-69** | 114.20 | 31.05 | 6.51 | 4.23 |
| 45 | **KM 16-75** | 60.40 | 52.25 | 5.51 | 5.25 |
| 46 | **KM 16-80** | 95.30 | 37.45 | 6.80 | 3.27 |
| 47 | **KM 16-82** | 107.25 | 25.40 | 5.96 | 2.86 |
| 48 | **KM 2241** | 89.35 | 18.60 | 7.29 | 2.34 |
| 49 | **KM 7-134** | 64.40 | 33.85 | 6.24 | 3.70 |
| 50 | **LGG 460** | 75.35 | 35.75 | 5.87 | 3.95 |
| 51 | **M 1032** | 68.35 | 24.90 | 7.80 | 3.15 |
| 52 | **M 1053** | 108.25 | 33.85 | 5.32 | 3.56 |
| 53 | **M 1131** | 80.35 | 42.50 | 8.01 | 3.26 |
| 54 | **M 1156** | 79.35 | 8.60 | 6.02 | 4.24 |
| 55 | **M 1168** | 53.00 | 30.70 | 7.25 | 4.21 |
| 56 | **M 1255** | 76.35 | 21.45 | 7.19 | 3.56 |
| 57 | **M 1370** | 75.35 | 27.40 | 7.23 | 3.67 |
| 58 | M 1372 | 94.30 | 41.40 | 14.60 | 2.66 |
| 59 | M 1378 | 63.40 | 29.95 | 7.37 | 4.19 |
| 60 | **M 1400** | 74.35 | 47.85 | 9.76 | 3.71 |
| 61 | **M 1447** | 56.40 | 30.40 | 6.88 | 3.48 |
| 62 | **M 145** | 56.40 | 50.65 | 5.24 | 4.05 |
| 63 | **M 1477** | 82.30 | 40.60 | 8.50 | 6.19 |
| 64 | **M 1485** | 61.40 | 41.85 | 6.08 | 5.40 |
| 65 | **M 1493** | 61.40 | 19.20 | 7.23 | 3.21 |
| 66 | **M 1503** | 61.40 | 18.50 | 4.81 | 2.55 |
| 67 | **M 204** | 61.40 | 23.80 | 7.09 | 4.20 |
| 68 | **M 289** | 62.40 | 24.00 | 7.64 | 5.23 |
| 69 | **M 313** | 54.90 | 38.25 | 6.57 | 2.80 |
| 70 | **M 422** | 73.35 | 8.90 | 6.76 | 3.74 |
| 71 | **M 499** | 93.30 | 40.15 | 4.72 | 5.33 |
| 72 | **M 678** | 76.35 | 38.10 | 6.47 | 4.11 |
| 73 | **M 684** | 58.40 | 34.95 | 7.29 | 3.67 |
| 74 | **M 700** | 77.35 | 31.65 | 4.72 | 4.94 |
| 75 | **M 765** | 56.40 | 12.05 | 5.87 | 5.50 |
| 76 | **M 837** | 88.30 | 36.20 | 6.24 | 5.43 |
| 77 | **M 875** | 67.35 | 36.70 | 7.52 | 2.89 |
| 78 | **M 880** | 65.40 | 35.25 | 8.34 | 3.74 |
| 79 | **M 906** | 70.35 | 48.75 | 6.90 | 3.56 |
| 80 | **M 958** | 72.35 | 9.05 | 6.53 | 3.04 |
| 81 | **M 961** | 98.90 | 17.20 | 7.52 | 4.23 |
| 82 | **M 981** | 83.30 | 34.95 | 8.48 | 3.67 |
| 83 | **M 989** | 58.40 | 39.20 | 7.15 | 3.56 |
| 84 | **M1129** | 63.40 | 18.95 | 11.28 | 5.25 |
| 85 | **M1209** | 75.35 | 33.10 | 4.19 | 2.48 |
| 86 | **M1334** | 83.30 | 44.05 | 8.01 | 4.50 |
| 87 | M1350 | 58.40 | 40.30 | 6.84 | 5.06 |
| 88 | **M1429** | 97.00 | 26.55 | 6.86 | 4.21 |
| 89 | **M1443** | 66.35 | 37.95 | 7.21 | 4.75 |
| 90 | **M322** | 56.40 | 28.70 | 8.89 | 5.46 |
| 91 | **M409** | 63.40 | 45.00 | 8.03 | 2.80 |
| 92 | **M450** | 62.40 | 37.30 | 6.49 | 4.25 |
| 93 | **M660** | 53.00 | 29.45 | 11.50 | 3.89 |
| 94 | **M-704** | 73.35 | 27.25 | 6.92 | 4.59 |
| 95 | **M729** | 65.40 | 37.15 | 8.38 | 2.56 |
| 96 | **M739** | 80.35 | 40.75 | 9.30 | 2.98 |
| 97 | **MH 1442** | 97.00 | 32.45 | 4.46 | 2.67 |
| 98 | **MH 215** | 101.80 | 30.25 | 7.66 | 5.29 |
| 99 | **MH 318** | 70.35 | 45.30 | 10.35 | 3.45 |
| 100 | **MH 565** | 92.30 | 38.45 | 6.43 | 5.68 |
| 101 | **MH 810** | 51.40 | 10.80 | 5.57 | 2.56 |
| 102 | **MH 934** | 69.35 | 15.65 | 8.09 | 2.67 |
| 103 | **MH 96-1** | 53.40 | 27.75 | 13.41 | 3.31 |
| 104 | **ML 1299** | 83.60 | 22.40 | 5.57 | 4.85 |
| 105 | **ML 1451** | 64.40 | 33.55 | 6.70 | 3.67 |
| 106 | **ML 1464** | 66.35 | 25.55 | 8.40 | 4.34 |
| 107 | **ML 1628** | 60.40 | 25.40 | 8.44 | 4.50 |
| 108 | **ML 2037** | 56.40 | 38.75 | 5.98 | 3.01 |
| 109 | **ML 818** | 121.20 | 30.90 | 7.48 | 3.63 |
| 110 | **MUSKAN** | 69.35 | 44.15 | 5.50 | 2.57 |
| 111 | **NM 1** | 53.40 | 21.30 | 10.35 | 3.31 |
| 112 | **OLRM 4** | 53.40 | 20.85 | 7.54 | 4.10 |
| 113 | **PDM 139** | 52.40 | 29.00 | 6.94 | 2.67 |
| 114 | **PLM 167** | 98.70 | 39.50 | 5.63 | 3.56 |
| 115 | **PLM 271** | 73.10 | 32.60 | 8.32 | 4.25 |
| 116 | **Prakash Nepal** | 69.35 | 43.30 | 6.04 | 3.88 |
| 117 | **PS 16** | 79.35 | 27.60 | 10.64 | 2.66 |
| 118 | **PUSA 0971** | 57.40 | 32.15 | 11.36 | 6.21 |
| 119 | **PUSA 1131** | 77.35 | 38.10 | 7.09 | 4.23 |
| 120 | **PUSA 1132** | 70.20 | 28.05 | 7.15 | 2.69 |
| 121 | **PUSA 1331** | 52.40 | 28.20 | 5.07 | 2.67 |
| 122 | **PUSA 1332** | 72.35 | 34.65 | 6.28 | 2.89 |
| 123 | **PUSA 1333** | 78.35 | 42.50 | 3.88 | 2.34 |
| 124 | **PUSA 1341** | 59.40 | 36.05 | 5.40 | 2.79 |
| 125 | **PUSA 1342** | 65.40 | 23.80 | 5.40 | 2.79 |
| 126 | **PUSA 1441** | 86.30 | 28.70 | 7.35 | 2.57 |
| 127 | **Pusa Ratna** | 82.30 | 33.25 | 8.01 | 3.33 |
| 128 | **Pusa Vishal** | 105.75 | 31.70 | 6.74 | 5.73 |
| 129 | **Pusa0871** | 71.35 | 25.85 | 9.00 | 2.99 |
| 130 | **Pusa Baisakhi** | 89.35 | 26.30 | 5.30 | 4.71 |
| 131 | **RMG 1028** | 63.40 | 42.35 | 8.79 | 4.06 |
| 132 | **RMG 1087** | 54.40 | 28.85 | 12.59 | 4.81 |
| 133 | **RMG 991** | 77.35 | 39.50 | 6.55 | 5.55 |
| 134 | **RMGP 1** | 74.35 | 19.90 | 6.80 | 4.30 |
| 135 | **SATYA** | 81.30 | 25.85 | 7.33 | 2.90 |
| 136 | **TM 96-2** | 85.30 | 28.20 | 5.87 | 2.94 |
| 137 | **TM 96-25** | 74.35 | 27.40 | 8.05 | 2.67 |
| 138 | **TM 9725** | 94.30 | 29.30 | 5.73 | 3.00 |
| 139 | **V 04-04** | 58.40 | 37.15 | 8.32 | 2.80 |
| 140 | **V 1109** | 94.30 | 36.85 | 7.44 | 4.11 |
| 141 | **V 1138** | 61.40 | 34.00 | 7.05 | 3.34 |
| 142 | **V 1153** | 74.35 | 32.15 | 14.85 | 2.78 |
| 143 | **V 3518** | 48.20 | 29.45 | 8.95 | 3.54 |
| 144 | **V 6183** | 85.30 | 42.20 | 7.44 | 2.56 |
| 145 | **Yellow Mung 1** | 62.40 | 48.75 | 11.87 | 3.21 |

**Supplementary Table 6. Pearson’s correlation coefficients between investigated traits**

|  | Iron (mg/Kg) | Zinc(mg/Kg) | Phytic Acid (mg/g) | Tannins (g/100g) |
| --- | --- | --- | --- | --- |
| Iron (mg/Kg) | 1 |  |  |  |
| Zinc(mg/Kg) | 0.077 | 1 |  |  |
| Phytic Acid (mg/g) | -0.288*** | -0.033 | 1 |  |
| Tannins (g/100g) | -0.027 | 0.065 | 0.013 | 1 |

*** indicates significant at p<0.05

**Supplementary Table 7. Principle component analysis of investigated traits**

| **Characters** | **PC1** | **PC2** | **PC3** | **PC4** |
| --- | --- | --- | --- | --- |
| Iron (mg/Kg) | -0.677 | 0.187 | 0.086 | -0.705 |
| Zinc (mg/Kg) | -0.345 | -0.601 | 0.673 | 0.254 |
| Phytic Acid (mg/g) | 0.636 | -0.272 | 0.326 | -0.643 |
| Tannins (g/100g) | -0.126 | -0.727 | -0.656 | -0.151 |
| Eigen Values | 1.318 | 1.104 | 0.876 | 0.701 |
| % Variance | 32.95 | 27.60 | 21.90 | 17.53 |
| Cumulative  %Variance | 32.95 | 60.55 | 82.46 | 100 |
| Most contributing traits | Phytic Acid (mg/g) | Iron (mg/Kg) | Zinc (mg/Kg)  Phytic Acid (mg/g) | Zinc (mg/Kg) |

PC1, principal component 1; PC2, principal component 2; PC3, principal component 3; PC4, principal component 4

**Supplementary Table 8. Details of SNPs and their corresponding genes associated with grain iron concentration**

| Sl. No | Trait | SNP ID | Position | Chromosome | Blink | | GLM | | Candidate genes | Gene description (as described in Legume Information System) |
| --- | --- | --- | --- | --- | --- | --- | --- | --- | --- | --- |
|  |  |  |  |  | -Log10(P value) | R square | -Log10(P value) | R square |  |  |
| 1 | Grain Iron | S9_7997249 | 7997249 | 9 | 3.778785 |  | 3.506442 | 0.135064 | *Vradi09g05530* | actin-related protein, regulation of actin filament polymerization |
|  |  |  |  |  |  |  |  |  | *Vradi09g05550* | Mitochondrial transcription termination factor family protein |
|  |  |  |  |  |  |  |  |  | *Vradi09g05540* | Homeobox-leucine zipper protein 3, sequence-specific DNA binding transcription factor activity |
|  |  |  |  |  |  |  |  |  | *Vradi09g05560* | FKBP-like peptidyl-prolyl cis-trans isomerase family protein |
|  |  |  |  |  |  |  |  |  | *Vradi09g05570* | Calcium-binding EF hand family protein |
|  |  |  |  |  |  |  |  |  | *Vradi09g05580* | protein prenyltransferase alpha subunit repeat-containing protein 1-like isoform X5 [*Glycine max* |
| 2 | Grain Iron | S4_18913871 | 18913871 | 4 | 3.687823 |  | 3.43155 | 0.132184 | *Vradi04g09970* | protein FLX-like 1-like isoform X1 [*Glycine max*] |
| 3 | Grain Iron | S1_17519079 | 17519079 | 1 | 3.510678 |  | 3.284414 | 0.126559 | *Vradi01g09630* | major intrinsic protein (MIP) family transporter |
|  |  |  |  |  |  |  |  |  | *Vradi01g09640* | NLI interacting factor-like phosphatase; |
|  |  |  |  |  |  |  |  |  | *Vradi01g09650* | apyrase 2 (hydrolase activity) |
| 4 | Grain Iron | S10_5415348 | 5415348 | 10 | 3.420367 |  | 3.208742 | 0.123685 | *Vradi10g01840* | ubiquitin carboxyl-terminal hydrolase-like protein |
|  |  |  |  |  |  |  |  |  | *Vradi10g01850* | ubiquitin carboxyl-terminal hydrolase-like protein |
|  |  |  |  |  |  |  |  |  | *Vradi10g01860* | mitochondrial import inner membrane translocase subunit Tim13 [*Glycine max*]; |
| 5 | Grain Iron | S7_54073179 | 54073179 | 7 | 3.380133 |  | 3.17 | 0.122403 | *Vradi07g30180* | tubulin folding cofactor B |
|  |  |  |  |  |  |  |  |  | *Vradi07g30190* | WRKY family transcription factor; |
|  |  |  |  |  |  |  |  |  | *Vradi07g30200* | SUMO-activating enzyme 1A |
|  |  |  |  |  |  |  |  |  | *Vradi07g30210* | squamosa promoter-binding-like protein 16-like [*Glycine max* |
|  |  |  |  |  |  |  |  |  | *Vradi07g30220* | gamma carbonic anhydrase like |
|  |  |  |  |  |  |  |  |  | *Vradi07g30230* | CBS domain-containing protein |
|  |  |  |  |  |  |  |  |  | *Vradi07g30240* | thaumatin-like protein 3 |
|  |  |  |  |  |  |  |  |  | *Vradi07g30250* | UDP-D-glucose/UDP-D-galactose 4-epimerase 1,UDP-glucose 4-epimerase activity, GO:0006012 (galactose metabolic process), GO:0044237 (cellular metabolic process), GO:0050662 (coenzyme binding) |
|  |  |  |  |  |  |  |  |  | *Vradi07g30260* | Calcium-dependent lipid-binding (CaLB domain) family protein |
|  |  |  |  |  |  |  |  |  | *Vradi07g30270* | methyl-CpG-binding domain-containing protein 13-like [*Glycine max* |
|  |  |  |  |  |  |  |  |  | *Vradi07g30280* | Transcription initiation factor TFIIE, beta subunit |
| 6 | Grain Iron | S3_6187525 | 6187525 | 3 | 3.287519 |  | 3.09 | 0.119449 | *Vradi03g04660* | F-box family protein |
|  |  |  |  |  |  |  |  |  | *Vradi03g04670* | RNA-binding protein 38-like isoform X3 [*Glycine max* |
|  |  |  |  |  |  |  |  |  | *Vradi03g04680* | uncharacterized protein LOC102670030 [*Glycine max*] ; |
|  |  |  |  |  |  |  |  |  | *Vradi03g04690* | GTP-binding signal recognition particle SRP54, G-domain n |
|  |  |  |  |  |  |  |  |  | *Vradi03g04700* | rho GTPase-activating protein 1-like [*Glycine max* |
|  |  |  |  |  |  |  |  |  | *Vradi03g04710* | Small nuclear ribonucleoprotein family protein |
|  |  |  |  |  |  |  |  |  | *Vradi03g04720* | SAUR-like auxin-responsive protein family |
|  |  |  |  |  |  |  |  |  | *Vradi03g04730* | ATP binding microtubule motor family protein isoform 1 n |
|  |  |  |  |  |  |  |  |  | *Vradi03g04740* | beta-fructofuranosidase; cell wall invertase I; fructosidase; Concanavalin A-like lectin/glucanases superfamily, carbohydrate metabolic process |
|  |  |  |  |  |  |  |  |  | *Vradi03g04750* | ubiquitin-60S ribosomal L40 fusion protein; , Zinc-binding ribosomal protein |
|  |  |  |  |  |  |  |  |  | *Vradi03g04760* | alpha/beta hydrolase domain-containing protein 11-like [*Glycine max* |
|  |  |  |  |  |  |  |  |  | *Vradi03g04770* | uncharacterized protein LOC102669414 [*Glycine max* |
| 7 | Grain Iron | S6_28880418 | 28880418 | 6 | 3.273497 |  | 3.08 | 0.119002 | *Vradi06g11960* | prolyl oligopeptidase family protein; IPR001375 (Peptidase S9, prolyl oligopeptidase, catalytic domain |
|  |  |  |  |  |  |  |  |  | *Vradi06g11970* | Peptidase S9 prolyl oligopeptidase active site domain protein n |
|  |  |  |  |  |  |  |  |  | *Vradi06g11980* | nuclear movement family protein |
|  |  |  |  |  |  |  |  |  | *Vradi06g11990* | Ypt/Rab-GAP domain of gyp1p superfamily protein |
|  |  |  |  |  |  |  |  |  | *Vradi06g12000* | ATP-dependent zinc metalloprotease FtsH-like |
| 8 | Grain Iron | S9_7660336 | 7660336 | 9 | 3.271048 |  | 3.082638 | 0.118923 | *Vradi09g05260* | Pentatricopeptide repeat (PPR-like) superfamily protein |
|  |  |  |  |  |  |  |  |  | *Vradi09g05270* | uncharacterized protein LOC100814035 [*Glycine max* |
|  |  |  |  |  |  |  |  |  | *Vradi09g05280* | metalloendoproteinase 1-like [*Glycine max*]; (metallopeptidase activity |
|  |  |  |  |  |  |  |  |  | *Vradi09g05290* | squamosa promoter-binding protein-like 12; |
|  |  |  |  |  |  |  |  |  | *Vradi09g05300* | squamosa promoter binding protein-like 8; |
|  |  |  |  |  |  |  |  |  | *Vradi09g05310* | uncharacterized protein LOC100306543 |
|  |  |  |  |  |  |  |  |  | *Vradi09g05320* | syntaxin-132-like [*Glycine max*]; IPR010989 (t-SNARE, vesicle-mediated transport |
| 9 | Grain Iron | S1_26049102 | 26049102 | 1 | 3.211427 |  | 3.03194 | 0.117019 | *Vradi01g12300* | receptor-like kinase, Leucine-rich repeat, |
|  |  |  |  |  |  |  |  |  | *Vradi01g12310* | Transducin/WD40 repeat-like superfamily protein |
| 10 | Grain Iron | S1_17519130 | 17519130 | 1 | 3.164476 |  | 2.991876 | 0.115518 | *Vradi01g09630* | major intrinsic protein (MIP) family transporter, Aquaporin-like; GO:0005215 (transporter activity), GO:0006810 (transport), GO:0016020 (membrane) |
|  |  |  |  |  |  |  |  |  | *Vradi01g09640* | NLI interacting factor-like phosphatase; |
|  |  |  |  |  |  |  |  |  | *Vradi01g09650* | apyrase 2 (hydrolase activity) |
|  |  |  |  |  |  |  |  |  | *Vradi01g09660* | apyrase 2 (hydrolase activity |
| 11 | Grain Iron | S1_34808082 | 34808082 | 1 | 3.147655 |  | 2.977492 | 0.114981 | *Vradi01g14120* | short-chain dehydrogenase TIC 32, chloroplastic-like [*Glycine max*] |
|  |  |  |  |  |  |  |  |  | *Vradi01g14130* | short-chain dehydrogenase TIC 32, chloroplastic-like [*Glycine max*] |
|  |  |  |  |  |  |  |  |  | *Vradi01g14140* | SKP1-like 21; IPR001232 (SKP1 component); GO:0006511 (ubiquitin-dependent protein catabolic process) |
|  |  |  |  |  |  |  |  |  | *Vradi01g14150* | uncharacterized protein LOC100778708 isoform X3 [*Glycine max*] |
|  |  |  |  |  |  |  |  |  | *Vradi01g14160* | C2H2-like zinc finger protein; (metal ion binding) |
|  |  |  |  |  |  |  |  |  | *Vradi01g14170* | ATP-binding microtubule motor family protein, putative, P-loop containing nucleoside triphosphate hydrolase) |
|  |  |  |  |  |  |  |  |  | *Vradi01g14180* | prolyl 4-hydroxylase subunit alpha-1-like [*Glycine max*]; GO:0005506 (iron ion binding), GO:0031418 (L-ascorbic acid binding), GO:0055114 (oxidation-reduction process) |
| 12 | Grain Iron | S9_7912942 | 7912942 | 9 | 3.090562 |  | 2.928553 | 0.113154 | *Vradi09g05420* | stress up-regulated Nod 19 protein; |
|  |  |  |  |  |  |  |  |  | *Vradi09g05430* | Phosphoglycerate mutase family protein; |
|  |  |  |  |  |  |  |  |  | *Vradi09g05440* | myb transcription factor |
|  |  |  |  |  |  |  |  |  | *Vradi09g05450* | Cytochrome P450 superfamily protein; GO:0005506 (iron ion binding), GO:0020037 (heme binding), GO:0055114 (oxidation-reduction process) |
|  |  |  |  |  |  |  |  |  | *Vradi09g05460* | UPF0481 protein At3g47200-like [*Glycine max*]; IPR004158 (Protein of unknown function DUF247, plant) |
|  |  |  |  |  |  |  |  |  | *Vradi09g05470* | UPF0481 protein At3g47200-like [*Glycine max*]; IPR004158 (Protein of unknown function)\| |
|  |  |  |  |  |  |  |  |  | *Vradi09g05480* | UPF0481 protein At3g47200-like [*Glycine max*]; IPR004158 (Protein of unknown function) |
|  |  |  |  |  |  |  |  |  | *Vradi09g05490* | stress up-regulated Nod 19 protein; IPR011692 (Stress up-regulated Nod 19) |
|  |  |  |  |  |  |  |  |  | *Vradi09g05500* | oxygen-evolving enhancer protein; IPR008797 (Photosystem II PsbQ, oxygen evolving complex), IPR023222 (PsbQ-like domain); GO:0005509 (calcium ion binding), GO:0009523 (photosystem II), GO:0009654 (photosystem II oxygen evolving complex), GO:0015979 (photosynthesis), GO:0019898 (extrinsic component of membrane) |
|  |  |  |  |  |  |  |  |  | *Vradi09g05510* | Mitochondrial transcription termination factor family protein; IPR003690 (Mitochodrial transcription termination factor-related); |
|  |  |  |  |  |  |  |  |  | *Vradi09g05520* | actin-related protein 2/3 complex subunit 5 |
|  |  |  |  |  |  |  |  |  | *Vradi09g05530* | actin-related protein 2/3 complex subunit 5; |
|  |  |  |  |  |  |  |  |  | *Vradi09g05540* | homeobox-leucine zipper protein 3; IPR003106 (Leucine zipper, homeobox-associated) |
|  |  |  |  |  |  |  |  |  | *Vradi09g05550* | Mitochondrial transcription termination factor family protein |
|  |  |  |  |  |  |  |  |  | *Vradi09g05560* | FKBP-like peptidyl-prolyl cis-trans isomerase family protein, GO:0006457 (protein folding) |
| 13 | Grain Iron | S2_20209632 | 20209632 | 2 | 3.038449 |  | 2.883721 | 0.111486 | *Vradi02g10980* | terpene synthase 21; IPR008930 (Terpenoid cyclases/protein prenyltransferase alpha-alpha toroid), IPR008949 (Terpenoid synthase); GO:0000287 (magnesium ion binding), GO:0008152 (metabolic process), GO:0010333 (terpene synthase activity), |
| 14 | Grain Iron | S1_28097267 | 28097267 | 1 | 3.019591 |  | 2.867461 | 0.110882 | *Vradi01g12660* | Glutathione S-transferase family protein |
| 15 | Grain Iron | S1_28097327 | 28097327 | 1 | 3.019591 |  | 2.867461 | 0.110882 | *Vradi01g12660* | Glutathione S-transferase family protein |

**Supplementary Table 9 Details of SNPs and their corresponding genes associated with grain zinc concentration**

| Sl. No | Trait | SNP ID | Position | Chromosome | Blink | | GLM | | Candidate genes | Gene description (as described in Legume Information System) |
| --- | --- | --- | --- | --- | --- | --- | --- | --- | --- | --- |
|  |  |  |  |  | -Log10(P value) | R square | R square | -Log10(P value) |  |  |
| 1 | Grain Zinc | S7_32662670 | 32662670 | 7 | 4.568692 |  | 0.151569 | 4.186573 | *Vradi07g13710* | adenylate cyclase |
| 2 | Grain Zinc | S5_33190115 | 33190115 | 5 | 3.895061 |  | 0.129957 | 3.64442 | *Vradi05g21740* | isoflavone reductase-like protein-like [*Glycine max*] |
|  |  |  |  |  |  |  |  |  | *Vradi05g21750* | N-terminal nucleophile aminohydrolases (Ntn hydrolases) superfamily protein; |
|  |  |  |  |  |  |  |  |  | *Vradi05g21760* | Pentatricopeptide repeat (PPR) superfamily protein |
|  |  |  |  |  |  |  |  |  | *Vradi05g21770* | COP9 signalosome complex subunit-like protein |
|  |  |  |  |  |  |  |  |  | *Vradi05g21780* | ceramide kinase-like protein, diacylglycerol kinase activity), GO:0007205 (protein kinase C-activating G-protein coupled receptor signaling pathway |
|  |  |  |  |  |  |  |  |  | *Vradi05g21790* | protein kinase family protein; Concanavalin A-like lectin/glucanase, subgroup), IPR014729 (Rossmann-like alpha/beta/alpha sandwich fold); GO:0000151 (ubiquitin ligase complex), GO:0004672 (protein kinase activity), GO:0004674 (protein serine/threonine kinase activity), GO:0004842 (ubiquitin-protein ligase activity), GO:0005524 (ATP binding), GO:0006468 (protein phosphorylation), GO:0016567 (protein ubiquitination) |
|  |  |  |  |  |  |  |  |  | *Vradi05g21800* | DERLIN-1 |
|  |  |  |  |  |  |  |  |  | *Vradi05g21810* | cell number regulator 6 [*Glycine max*]; |
| 3 | Grain Zinc | S5_33190129 | 33190129 | 5 | 3.895061 |  | 0.129957 | 3.64442 | *NA* | NA |
| 4 | Grain Zinc | S10_11495079 | 11495079 | 10 | 3.520373 |  | 0.117792 | 3.332246 | *Vradi10g04820* | sucrose nonfermenting 4-like protein-like isoform 1 [*Glycine max*]; adenyl nucleotide binding |
|  |  |  |  |  |  |  |  |  | *Vradi10g04830* | zinc finger (Ran-binding) family protein; GO:0008270 (zinc ion binding) |
|  |  |  |  |  |  |  |  |  | *Vradi10g04840* | DNA binding;nucleotide binding;nucleic acid binding;DNA-directed DNA polymerases;DNA-directed DNA polymerases; C4-type zinc-finger of DNA polymerase delta |
|  |  |  |  |  |  |  |  |  | *Vradi10g04850* | Protein kinase superfamily protein; GO:0006468 (protein phosphorylation) |
|  |  |  |  |  |  |  |  |  | *Vradi10g04860* | microtubule-binding protein TANGLED-like [*Glycine max*] |
|  |  |  |  |  |  |  |  |  | *Vradi10g04870* | Xaa-pro aminopeptidase P, GO:0016787 (hydrolase activity) |
|  |  |  |  |  |  |  |  |  | *Vradi10g04880* | protein AUXIN RESPONSE 4-like [*Glycine max*] |
|  |  |  |  |  |  |  |  |  | *Vradi10g04890* | Pentatricopeptide repeat (PPR) superfamily protein |
|  |  |  |  |  |  |  |  |  | *Vradi10g04900* | Eukaryotic aspartyl protease family protein; |
| 5 | Grain Zinc | S5_26040601 | 26040601 | 5 | 3.212871 |  | 0.1077 | 3.070178 | *Vradi05g17000* | F-box family protein |
|  |  |  |  |  |  |  |  |  | *Vradi05g17010* | polygalacturonase 4; GO:0005975 (carbohydrate metabolic process) |
|  |  |  |  |  |  |  |  |  | *Vradi05g17020* | actin-related protein, GO:0032502 (developmental process) |
|  |  |  |  |  |  |  |  |  | *Vradi05g17030* | magnesium ion binding;thiamin pyrophosphate binding;hydro-lyases;catalytics;2-succinyl-5-enolpyruvyl- 6-hydroxy-3-cyclohexene-1-carboxylic-acid synthases; GO:0000287 (magnesium ion binding) |
|  |  |  |  |  |  |  |  |  | *Vradi05g17040* | RNA polymerase sigma factor; |
|  |  |  |  |  |  |  |  |  | *Vradi05g17050* | alpha-N-acetylglucosaminidase family protein |
|  |  |  |  |  |  |  |  |  | *Vradi05g17060* | polyamine oxidase 1; GO:0016491 (oxidoreductase activity) |
|  |  |  |  |  |  |  |  |  | *Vradi05g17070* | Copper amine oxidase family protein; GO:0005507 (copper ion binding), GO:0008131 (primary amine oxidase activity), GO:0009308 (amine metabolic process), GO:0048038 (quinone binding), GO:0055114 (oxidation-reduction process); |
|  |  |  |  |  |  |  |  |  | *Vradi05g17080* | RNA-binding KH domain-containing protein |
| 6 | Grain Zinc | S5_25508831 | 25508831 | 5 | 3.13314 |  | 0.10513 | 3.001348 | *Vradi05g16640* | DNAJ heat shock family protein; IPR012724 (Chaperone DnaJ); GO:0005524 (ATP binding), GO:0006457 (protein folding), GO:0009408 (response to heat), GO:0031072 (heat shock protein binding), |
|  |  |  |  |  |  |  |  |  | *Vradi05g16650* | unknown protein |
|  |  |  |  |  |  |  |  |  | *Vradi05g16660* | Signal recognition particle, GO:0006614 (SRP-dependent cotranslational protein targeting to membrane), GO:0017111 (nucleoside-triphosphatase activity), GO:0048500 (signal recognition particle) |
|  |  |  |  |  |  |  |  |  | *Vradi05g16670* | Phox (PX) domain-containing protein; GO:0035091 (phosphatidylinositol binding) |
|  |  |  |  |  |  |  |  |  | *Vradi05g16680* | formamidopyrimidine-DNA glycosylase, GO:0008270 (zinc ion binding) |
|  |  |  |  |  |  |  |  |  | *Vradi05g16690* | NADH-ubiquinone oxidoreductase 24 kDa subunit, putative |
|  |  |  |  |  |  |  |  |  | *Vradi05g16700* | Calcium-binding EF-hand family protein; GO:0005509 (calcium ion binding) |
|  |  |  |  |  |  |  |  |  | *Vradi05g16710* | signal peptidase complex catalytic subunit |
|  |  |  |  |  |  |  |  |  | *Vradi05g16720* | Pentatricopeptide repeat (PPR) superfamily protein |
|  |  |  |  |  |  |  |  |  | *Vradi05g16730* | major intrinsic protein (MIP) family transporter; IPR000425 (Major intrinsic protein), IPR023271 (Aquaporin-like); GO:0005215 (transporter activity), GO:0006810 (transport), GO:0016020 (membrane) |
| 7 | Grain Zinc | S5_33190141 | 33190141 | 5 | 3.055024 |  | 0.102567 | 2.93 | *Vradi05g21740* | isoflavone reductase-like protein-like [*Glycine max*] |
|  |  |  |  |  |  |  |  |  | *Vradi05g21750* | N-terminal nucleophile aminohydrolases (Ntn hydrolases) superfamily protein |
|  |  |  |  |  |  |  |  |  | *Vradi05g21760* | Pentatricopeptide repeat (PPR) superfamily protein |
|  |  |  |  |  |  |  |  |  | *Vradi05g21770* | COP9 signalosome complex subunit-like protein |
|  |  |  |  |  |  |  |  |  | *Vradi05g21780* | ceramide kinase-like protein |
|  |  |  |  |  |  |  |  |  | *Vradi05g21790* | protein kinase family protein |
|  |  |  |  |  |  |  |  |  | *Vradi05g21800* | DERLIN-1 |
|  |  |  |  |  |  |  |  |  | *Vradi05g21810* | cell number regulator 6 |
|  |  |  |  |  |  |  |  |  | *Vradi05g21820* | F-box family protein |
|  |  |  |  |  |  |  |  |  | *Vradi05g21830* | folate/biopterin transporter |
| 8 | Grain Zinc | S7_21629408 | 21629408 | 7 | 3.050153 |  | 0.1024 | 2.929 | *NA* | NA |
| 9 | Grain Zinc | S7_21629431 | 21629431 | 7 | 3.050153 |  | 0.1024 | 2.929 | *NA* | NA |
| 10 | Grain Zinc | S2_692564 | 692564 | 2 | 3.032915 |  | 0.1018 | 2.91 | *Vradi02g00640* | structural maintenance of chromosomes 6A |
|  |  |  |  |  |  |  |  |  | *Vradi02g00650* | F-box family protein |
|  |  |  |  |  |  |  |  |  | *Vradi02g00660* | 2-phosphoglycerate kinase |
|  |  |  |  |  |  |  |  |  | *Vradi02g00670* | aminoalcoholphosphotransferase 1 |
|  |  |  |  |  |  |  |  |  | *Vradi02g00680* | aldo/keto reductase family oxidoreductase; (oxidation-reduction process |
|  |  |  |  |  |  |  |  |  | *Vradi02g00690* | receptor-like protein kinase 2 |
|  |  |  |  |  |  |  |  |  | *Vradi02g00700* | Small nuclear ribonucleoprotein family protein |
|  |  |  |  |  |  |  |  |  | *Vradi02g00710* | vacuolar fusion protein CCZ1 homolog B-like isoform X1 [*Glycine max*] |
|  |  |  |  |  |  |  |  |  | *Vradi02g00720* | cytokinin riboside 5'-monophosphate phosphoribohydrolase LOG1 [*Glycine max*] |
|  |  |  |  |  |  |  |  |  | *Vradi02g00730* | actin-related protein 7; GO:0032502 (developmental process) |
|  |  |  |  |  |  |  |  |  | *Vradi02g00740* | uncharacterized protein LOC100810497 [*Glycine max*] |
|  |  |  |  |  |  |  |  |  | *Vradi02g00750* | tRNA-dihydrouridine synthase |

**Supplementary Table 10 Details of SNPs and their corresponding genes associated with grain phytic acid content**

| Sl. No | Trait | SNP ID | Position | Chromosome | Blink | | GLM | | Candidate genes | Gene description (as described in Legume Information System) |
| --- | --- | --- | --- | --- | --- | --- | --- | --- | --- | --- |
|  |  |  |  |  | -Log10(P value) | R square | -Log10(P value) | R square |  |  |
| 1 | Grain Phytic acid | S8_27069506 | 27069506 | 8 | 5.306323 | 0.179106 | 0.187073 | 4.709468 | *Vradi08g09870* | Serine/threonine protein phosphatase family protein |
|  |  |  |  |  |  |  |  |  | *Vradi08g09880* | U3 small nucleolar RNA-associated protein 15 homolog isoform X2 [*Glycine max* |
|  |  |  |  |  |  |  |  |  | *Vradi08g09890* | peroxidase 3-like [*Glycine max*]; IPR010255 (Haem peroxidase), GO:0006979 (response to oxidative stress), GO:0020037 (heme binding), GO:0055114 (oxidation-reduction process) |
| 2 | Grain Phytic acid | S8_27209879 | 27209879 | 8 | 5.090314 | 0.169824 | 0.180499 | 4.547483 | *Vradi08g09970* | tubby like protein 3; IPR001810 (F-box domain), IPR025659 (Tubby C-terminal-like domain) |
| 3 | Grain Phytic acid | S2_22069747 | 22069747 | 2 | 4.600703 | 0.153407 | 0.165452 | 4.171866 | *Vradi02g11650* | ATP binding/protein serine/threonine kinase [*Glycine max*] |
|  |  |  |  |  |  |  |  |  | *Vradi02g11660* | Serine/Threonine kinase family protein; |
|  |  |  |  |  |  |  |  |  | *Vradi02g11670* | Transmembrane amino acid transporter family protein; IPR013057 (Amino acid transporter, transmembrane) |
|  |  |  |  |  |  |  |  |  | *Vradi02g11680* | MADS-box transcription factor |
|  |  |  |  |  |  |  |  |  | *Vradi02g11690* | uncharacterized protein |
|  |  |  |  |  |  |  |  |  | *Vradi02g11700* | uncharacterized protein |
|  |  |  |  |  |  |  |  |  | *Vradi02g11710* | calmodulin-binding transcription activator 4-like isoform X1 |
| 4 | Grain Phytic acid | S2_22069711 | 22069711 | 2 | 4.600703 | 0.153407 | 0.163001 | 4.110043 | *Vradi02g11650* | ATP binding/protein serine/threonine kinase |
|  |  |  |  |  |  |  |  |  | *Vradi02g11660* | Serine/Threonine kinase family protein; |
|  |  |  |  |  |  |  |  |  | *Vradi02g11670* | Transmembrane amino acid transporter family protein |
|  |  |  |  |  |  |  |  |  | *Vradi02g11680* | MADS-box transcription factor; |
|  |  |  |  |  |  |  |  |  | *Vradi02g11690* | uncharacterized protein |
|  |  |  |  |  |  |  |  |  | *Vradi02g11700* | uncharacterized protein |
|  |  |  |  |  |  |  |  |  | *Vradi02g11710* | calmodulin-binding transcription activator 4-like isoform X1 |
| 5 | Grain Phytic acid | S1_21547083 | 21547083 | 1 | 4.521586 | 0.134642 | 0.154128 | 3.88453 | *Vradi01g11110* | Phosphatidyl inositol kinase (PIK-G1) n |
|  |  |  |  |  |  |  |  |  | *Vradi01g11070* | transcription factor HEC3-like |
|  |  |  |  |  |  |  |  |  | *Vradi01g11080* | protein YLS7-like |
|  |  |  |  |  |  |  |  |  | *Vradi01g11090* | chromatin assembly factor 1 subunit FAS2-like isoform X1 |
|  |  |  |  |  |  |  |  |  | *Vradi01g11100* | Cytochrome P450 superfamily protein; domain); activity), GO:0005506 (iron ion binding), GO:0020037 (heme binding), |
|  |  |  |  |  |  |  |  |  | *Vradi01g11120* | NAC domain containing protein 103; |
|  |  |  |  |  |  |  |  |  | *Vradi01g11130* | BTB/POZ domain-containing protein ; |
|  |  |  |  |  |  |  |  |  | *Vradi01g11140* | Coiled-coil domain-containing protein 18, putative isoform 1 n |
|  |  |  |  |  |  |  |  |  | *Vradi01g11150* | callose synthase 5 |

**Supplementary Table 11. Details of SNPs and their corresponding genes associated with grain tannin content**

| Sl. No | Trait | SNP ID | Position | Chromosome | Blink | | GLM | | Candidate genes | Gene description (as described in Legume Information System) |
| --- | --- | --- | --- | --- | --- | --- | --- | --- | --- | --- |
|  |  |  |  |  | -Log10(P value) | R square | -Log10(P value) | R square |  |  |
| 1 | Grain Tannin | S6_35205606 | 35205606 | 6 | 4.404338 |  | 0.12448 | 3.566654 | *Vradi06g15070* | SBP (S-ribonuclease binding protein) family protein |
|  |  |  |  |  |  |  |  |  | *Vradi06g15080* | serine/threonine-protein phosphatase 7 long form homolog |
|  |  |  |  |  |  |  |  |  | *Vradi06g15090* | heat shock transcription factor B4 |
|  |  |  |  |  |  |  |  |  | *Vradi06g15100* | triacylglycerol lipase-like 1; GO:0006629 (lipid metabolic process) |
|  |  |  |  |  |  |  |  |  | *Vradi06g15110* | remorin-like protein |
|  |  |  |  |  |  |  |  |  | *Vradi06g15120* | RNA cap guanine-N2 methyltransferase; GO:0008168 (methyltransferase activity), GO:0009452 (7-methylguanosine RNA capping) |
|  |  |  |  |  |  |  |  |  | *Vradi06g15130* | THO complex, subunit 5 |
|  |  |  |  |  |  |  |  |  | *Vradi06g15140* | glutamate receptor 2.5; IPR001638 (Extracellular solute-binding protein, family 3) |
|  |  |  |  |  |  |  |  |  | *Vradi06g15150* | Protein kinase family protein; GO:0004674 (protein serine/threonine kinase activity) |
|  |  |  |  |  |  |  |  |  | *Vradi06g15160* | uncharacterized protein |
| 2 | Grain Tannin | S6_35205625 | 35205625 | 6 | 4.404338 |  | 0.12448 | 3.566654 | *Vradi06g15070* | SBP (S-ribonuclease binding protein) family protein |
|  |  |  |  |  |  |  |  |  | *Vradi06g15080* | serine/threonine-protein phosphatase 7 long form homolog |
|  |  |  |  |  |  |  |  |  | *Vradi06g15090* | heat shock transcription factor B4 |
|  |  |  |  |  |  |  |  |  | *Vradi06g15100* | triacylglycerol lipase-like 1; GO:0006629 (lipid metabolic process) |
|  |  |  |  |  |  |  |  |  | *Vradi06g15110* | remorin-like protein |
|  |  |  |  |  |  |  |  |  | *Vradi06g15120* | RNA cap guanine-N2 methyltransferase |
|  |  |  |  |  |  |  |  |  | *Vradi06g15130* | THO complex |
|  |  |  |  |  |  |  |  |  | *Vradi06g15140* | glutamate receptor 2.5; IPR001638 (Extracellular solute-binding protein, family 3) |
|  |  |  |  |  |  |  |  |  | *Vradi06g15150* | Protein kinase family protein; GO:0004674 (protein serine/threonine kinase activity) |
|  |  |  |  |  |  |  |  |  | *Vradi06g15160* | uncharacterized protein |
| 3 | Grain Tannin | S4_20722213 | 20722213 | 4 | 4.243338 |  | 0.119959 | 3.45108 | *Vradi04g11540* | SIGNAL PEPTIDE PEPTIDASE-LIKE 5 |
|  |  |  |  |  |  |  |  |  | *Vradi04g11550* | Pollen Ole e 1 allergen and extensin family protein |
|  |  |  |  |  |  |  |  |  | *Vradi04g11560* | SAUR-like auxin-responsive protein family |
|  |  |  |  |  |  |  |  |  | *Vradi04g11570* | Protein kinase superfamily protein; GO:0004674 (protein serine/threonine kinase activity) |
|  |  |  |  |  |  |  |  |  | *Vradi04g11580* | Sugar transporter SWEET n |
|  |  |  |  |  |  |  |  |  | *Vradi04g11590* | Sugar transporter SWEET n |
| 4 | Grain Tannin | S9_5005061 | 5005061 | 9 | 4.155195 |  | 0.117478 | 3.387353 | NA | NA |
| 5 | Grain Tannin | S7_35555182 | 35555182 | 7 | 4.001369 |  | 0.112727776 | 3.264701572 | *Vradi07g15310* | Nitrate transporter, Proton-dependent oligopeptide transporter family |
