# Supplementary figures and images for: Genome wide association analysis for grain micronutrients and anti-nutritional traits in mungbean [Vigna radiata (L.) R. Wilczek] using SNP markers
Source: Front Nutr. 2023 Feb 7;10:1099004. doi: 10.3389/fnut.2023.1099004 (PMC9941709; doi:10.3389/fnut.2023.1099004)

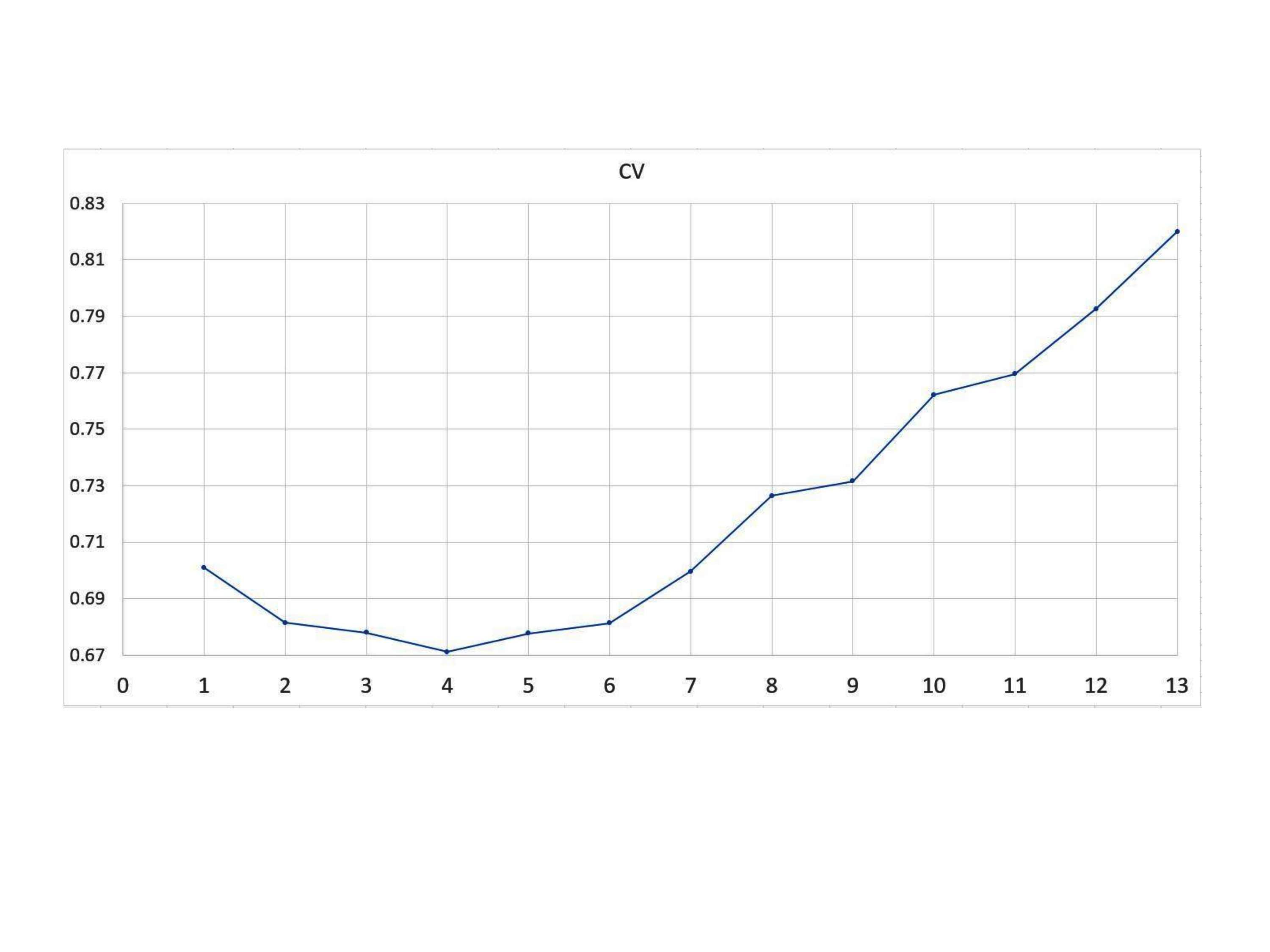

Supplement: Supplementary Figure 1 — Determination of Q matrix with the lowest cross-validation error (in this case k = 4) (X-axis has the values of k while the Y-axis contains the values of CV at corresponding k). [file Image_1.JPEG]

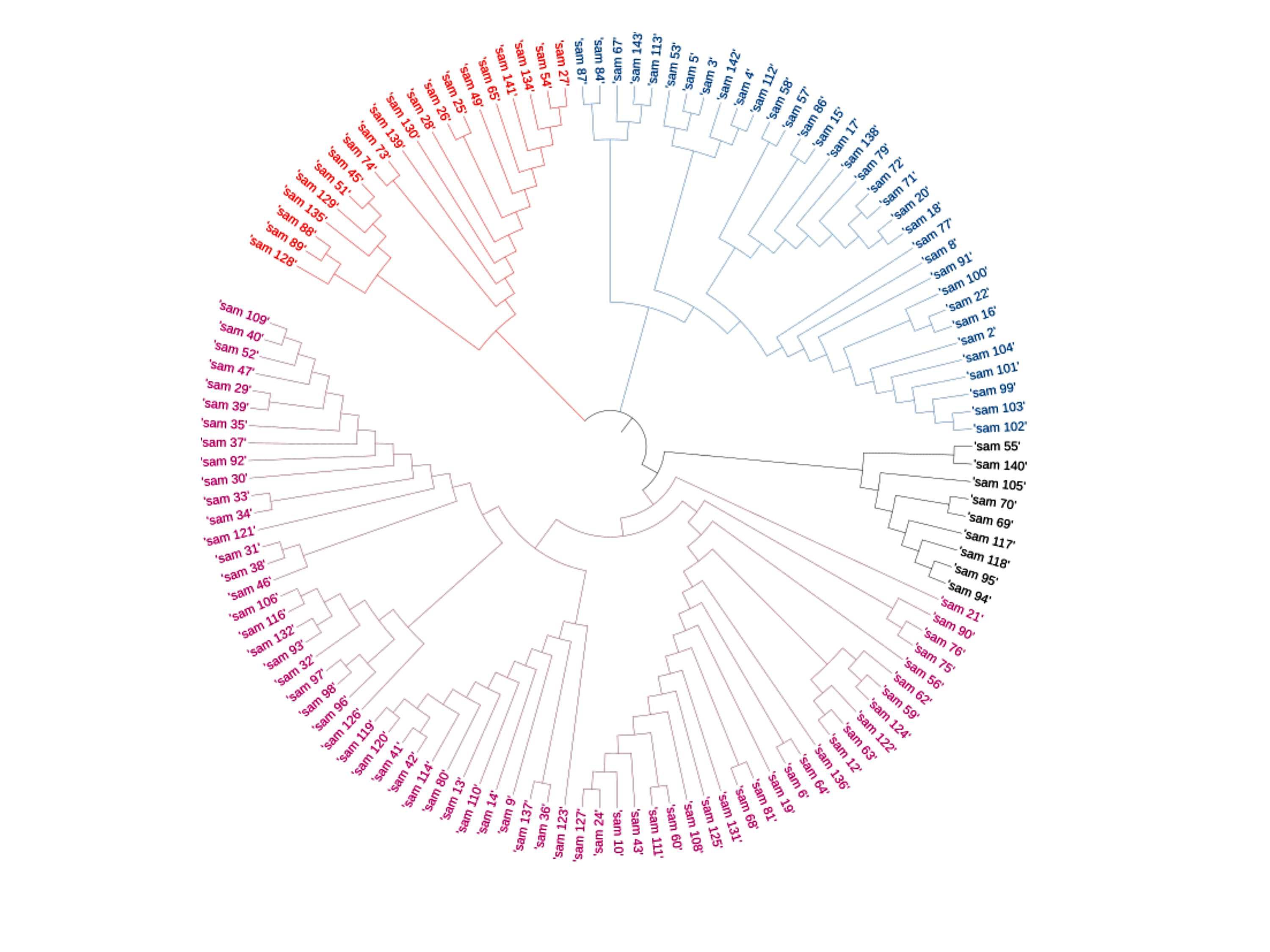

Supplement: Supplementary Figure 2 — Phylogenetic tree depicting the genetic relations among 127 diverse mungbean genotypes based on Nei’s genetic distance using 14,447 high quality GBS based SNPs. [file Image_2.JPEG]

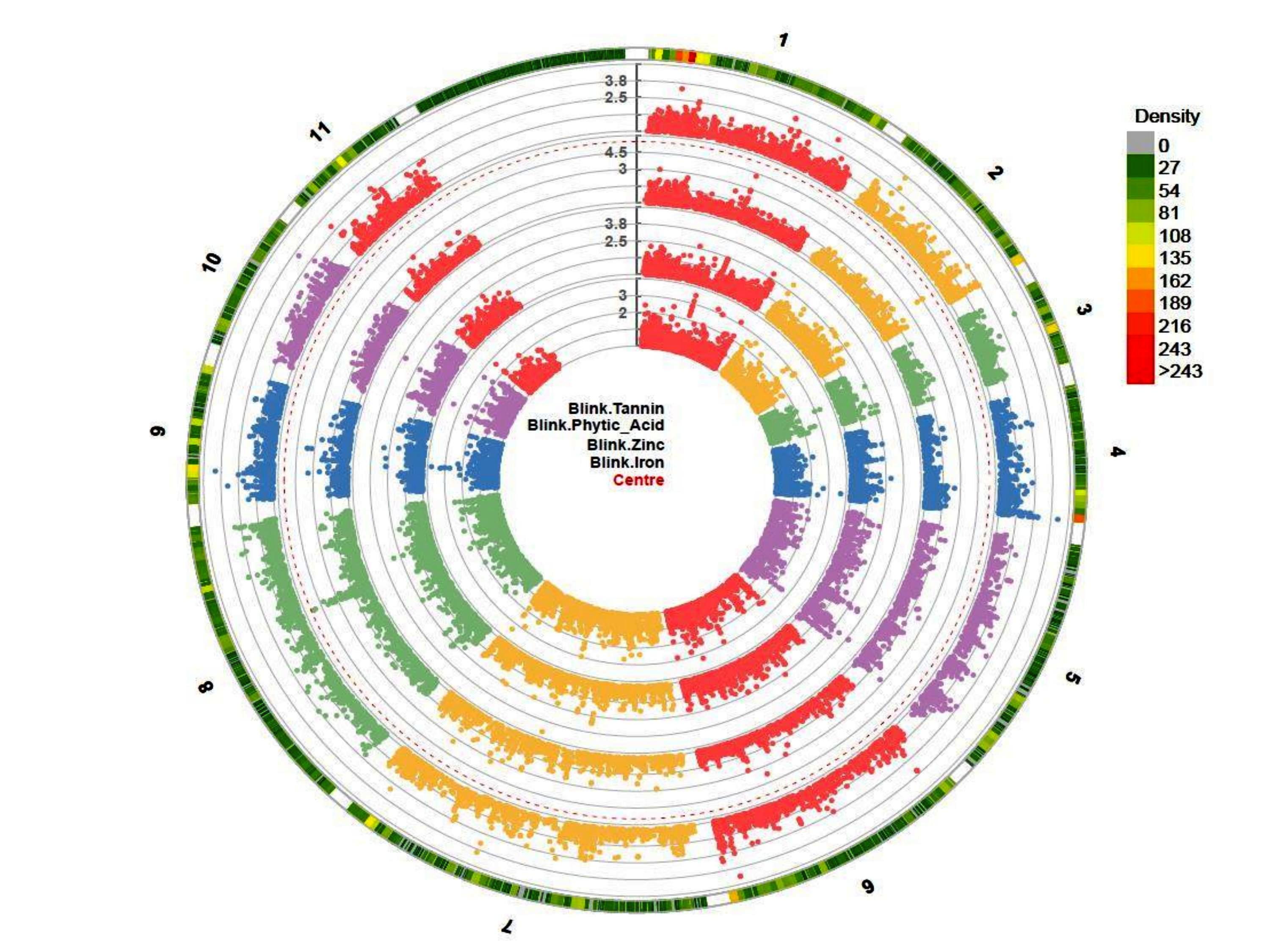

Supplement: Supplementary Figure 3 — Circular diagram depicting a summarized view of the significant association of SNP markers with all the four traits in the study along with SNP density in the outer ring. [file Image_3.JPEG]
